# Supplementary material for: Triterpene Derivatives from Garcinia oligantha and Their Anti-Cancer Activity
Source: Plants (Basel). 2023 Jan 3;12(1):192. doi: 10.3390/plants12010192 (PMC9824146; doi:10.3390/plants12010192)
Supplement: Supplementary file 1 [file plants-12-00192-s001.zip › plants-2083759-supplementary.pdf]

Content Page

Figure S1	^{1}H NMR (600 MHz, $CDCl_3$) spectrum of compound **1**	3
Figure S2	Enlarged ^{1}H NMR (600 MHz, $CDCl_3$) spectrum of compound **1**	3
Figure S3	^{13}C NMR (150 MHz, $CDCl_3$) spectrum of compound **1**	4
Figure S4	Enlarged ^{13}C NMR (150 MHz, $CDCl_3$) spectrum of compound **1**	4
Figure S5	HSQC ($CDCl_3$) spectrum of compound **1**	5
Figure S6	Enlarged HSQC ($CDCl_3$) spectrum of compound **1**	5
Figure S7	HMBC ($CDCl_3$) spectrum of compound **1**	6
Figure S8	Enlarged HMBC ($CDCl_3$) spectrum of compound **1**	6
Figure S9	NOESY ($CDCl_3$) spectrum of compound **1**	7
Figure S10	HRESIMS spectrum of compound **1**	7
Figure S11	^{1}H NMR (600 MHz, $CDCl_3$) spectrum of compound **1a**	8
Figure S12	^{13}C NMR (150 MHz, $CDCl_3$) spectrum of compound **1a**	8
Figure S13	^{1}H NMR (600 MHz, $CDCl_3$) spectrum of compound **2**	9
Figure S14	Enlarged ^{1}H NMR (600 MHz, $CDCl_3$) spectrum of compound **2**	9
Figure S15	^{13}C NMR (150 MHz, $CDCl_3$) spectrum of compound **2**	10
Figure S16	Enlarged ^{13}C NMR (150 MHz, $CDCl_3$) spectrum of compound **2**	10
Figure S17	HSQC ($CDCl_3$) spectrum of compound **2**	11
Figure S18	Enlarged HSQC ($CDCl_3$) spectrum of compound **2**	11
Figure S19	HMBC ($CDCl_3$) spectrum of compound **2**	12
Figure S20	Enlarged HMBC ($CDCl_3$) spectrum of compound **2**	12
Figure S21	NOESY ($CDCl_3$) spectrum of compound **2**	13
Figure S22	HRESIMS spectrum of compound **2**	13
Figure S23	^{1}H NMR (600 MHz, CD_3OD) spectrum of compound **3**	14
Figure S24	Enlarged ^{1}H NMR (600 MHz, CD_3OD) spectrum of compound **3**	14
Figure S25	^{13}C NMR (150 MHz, CD_3OD) spectrum of compound **3**	15
Figure S26	Enlarged ^{13}C NMR (150 MHz, CD_3OD) spectrum of compound **3**	15
Figure S27	^{13}C NMR (150 MHz, CD3Cl) spectrum of compound **3**	16
Figure S28	HSQC (CD_3OD) spectrum of compound **3**	16
Figure S29	Enlarged HSQC (CD_3OD) spectrum of compound **3**	17
Figure S30	HMBC (CD_3OD) spectrum of compound **3**	17
Figure S31	Enlarged HMBC (CD_3OD) spectrum of compound **3**	18
Figure S32	NOESY (CD_3OD) spectrum of compound **3**	18
Figure S33	HRESIMS spectrum of compound **3**	19
Figure S34	^{1}H NMR (600 MHz, CD_3OD) spectrum of compound **4**	19
Figure S35	Enlarged ^{1}H NMR (600 MHz, CD_3OD) spectrum of compound **4**	20
Figure S36	^{13}C NMR (150 MHz, CD_3OD) spectrum of compound **4**	20
Figure S37	Enlarged ^{13}C NMR (150 MHz, CD_3OD) spectrum of compound **4**	21
Figure S38	HSQC (CD_3OD) spectrum of compound **4**	21
Figure S39	Enlarged HSQC (CD_3OD) spectrum of compound **4**	22
Figure S40	HMBC (CD_3OD) spectrum of compound **4**	22
Figure S41	Enlarged HMBC (CD_3OD) spectrum of compound **4**	23
Figure S42	NOESY (CD_3OD) spectrum of compound **4**	23
Figure S43	HRESIMS spectrum of compound **4**	24

Figure S44	^{1}H NMR (600 MHz, CD_3OD) spectrum of compound **5**	24
Figure S45	Enlarged ^{1}H NMR (600 MHz, CD_3OD) spectrum of compound **5**	25
Figure S46	^{13}C NMR (150 MHz, CD_3OD) spectrum of compound **5**	25
Figure S47	Enlarged ^{13}C NMR (150 MHz, CD_3OD) spectrum of compound **5**	26
Figure S48	HSQC (CD_3OD) spectrum of compound **5**	26
Figure S49	Enlarged HSQC (CD_3OD) spectrum of compound **5**	27
Figure S50	HMBC (CD_3OD) spectrum of compound **5**	27
Figure S51	Enlarged HMBC (CD_3OD) spectrum of compound **5**	28
Figure S52	NOESY (CD_3OD) spectrum of compound **5**	28
Figure S53	HRESIMS spectrum of compound **5**	29
Figure S54	^{1}H NMR (600 MHz, $CDCl_3$) spectrum of compound **6**	29
Figure S55	Enlarged ^{1}H NMR (600 MHz, $CDCl_3$) spectrum of compound **6**	30
Figure S56	^{13}C NMR (150 MHz, $CDCl_3$) spectrum of compound **6**	30
Figure S57	Enlarged ^{13}C NMR (150 MHz, $CDCl_3$) spectrum of compound **6**	31
Figure S58	^{1}H NMR (600 MHz, $CDCl_3$) spectrum of compound **7**	31
Figure S59	Enlarged ^{1}H NMR (600 MHz, $CDCl_3$) spectrum of compound **7**	32
Figure S60	^{13}C NMR (150 MHz, $CDCl_3$) spectrum of compound **7**	32
Figure S61	Enlarged ^{13}C NMR (150 MHz, $CDCl_3$) spectrum of compound **7**	33
Figure S62	^{1}H NMR (600 MHz, CD_3OD) spectrum of compound **8**	33
Figure S63	Enlarged ^{1}H NMR (600 MHz, CD_3OD) spectrum of compound **8**	34
Figure S64	^{13}C NMR (150 MHz, CD_3OD) spectrum of compound **8**	34
Figure S65	Enlarged ^{13}C NMR (150 MHz, CD_3OD) spectrum of compound **8**	35
Figure S66	^{1}H NMR (600 MHz, CD_3OD) spectrum of compound **9**	35
Figure S67	Enlarged ^{1}H NMR (600 MHz, CD_3OD) spectrum of compound **9**	36
Figure S68	^{13}C NMR (150 MHz, CD_3OD) spectrum of compound **9**	36
Figure S69	Enlarged ^{13}C NMR (150 MHz, CD_3OD) spectrum of compound **9**	37
Figure S70	^{1}H NMR (600 MHz, CD_3OD) spectrum of compound **10**	37
Figure S71	Enlarged ^{1}H NMR (600 MHz, CD_3OD) spectrum of compound **10**	38
Figure S72	^{13}C NMR (150 MHz, CD_3OD) spectrum of compound **10**	38
Figure S73	Enlarged ^{13}C NMR (150 MHz, CD_3OD) spectrum of compound **10**	39
Figure S74	^{1}H NMR (600 MHz, CD_3OD) spectrum of compound **11**	39
Figure S75	Enlarged ^{1}H NMR (600 MHz, CD_3OD) spectrum of compound **11**	40
Figure S76	^{13}C NMR (150 MHz, CD_3OD) spectrum of compound **11**	40
Figure S77	Enlarged ^{13}C NMR (150 MHz, CD_3OD) spectrum of compound **11**	41
Figure S78	Extraction and separation flow chart	42

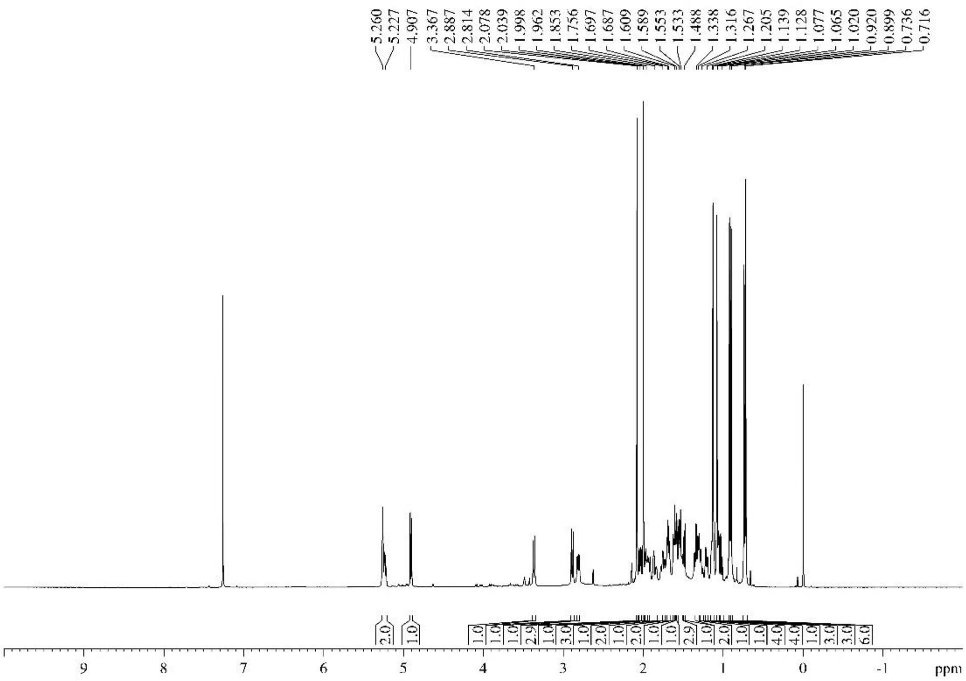

Figure S1. ^{1}H NMR (600 MHz, $CDCl_3$) spectrum of compound **1**

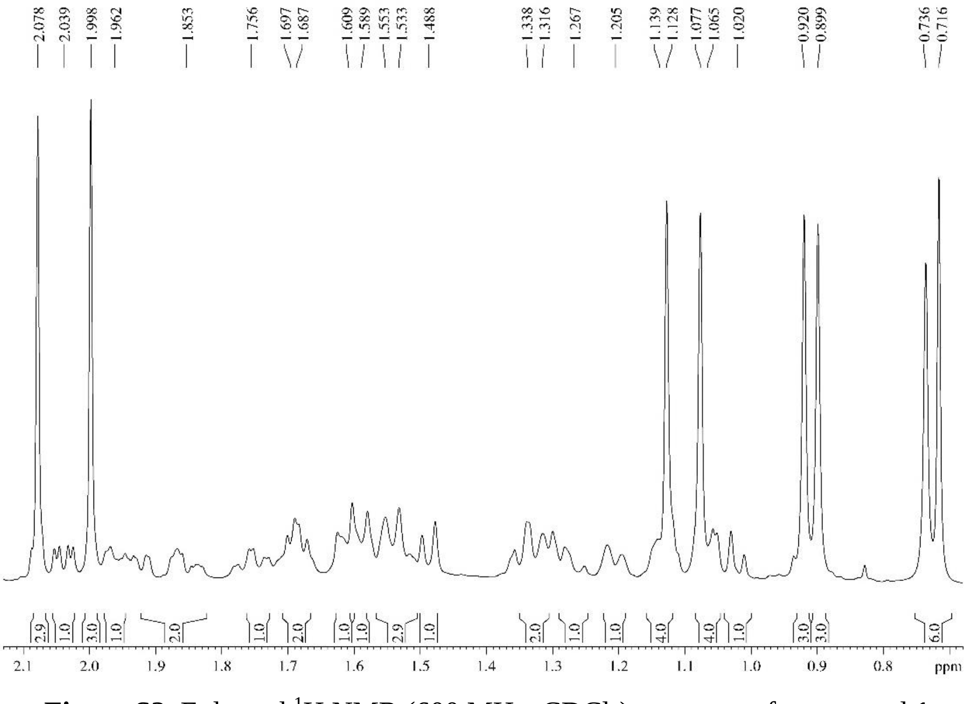

Figure S2. Enlarged ^{1}H NMR (600 MHz, $CDCl_3$) spectrum of compound **1**

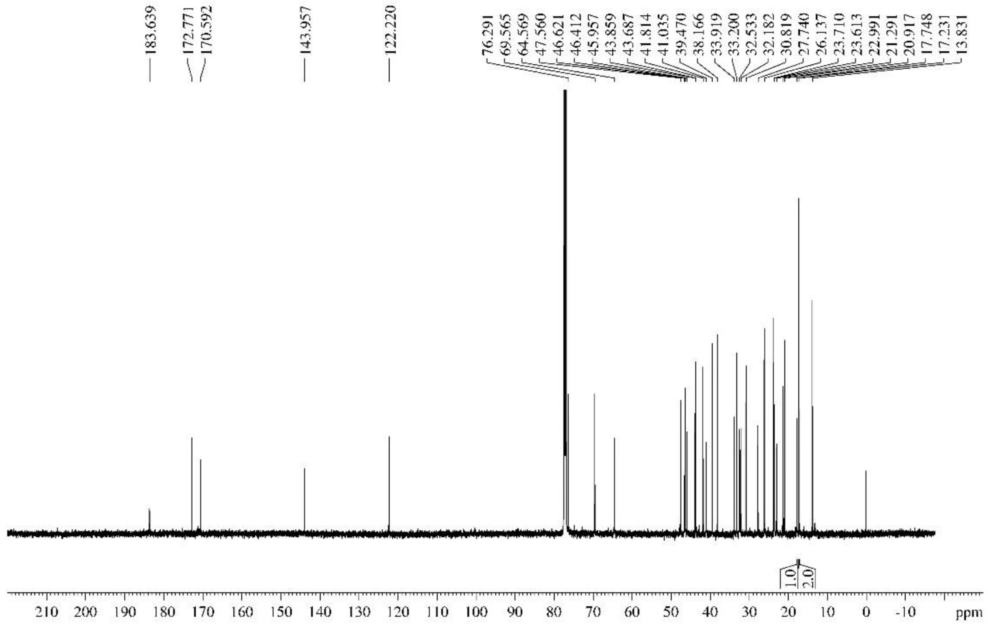

Figure S3. ^{13}C NMR (150 MHz, $CDCl_3$) spectrum of compound **1**

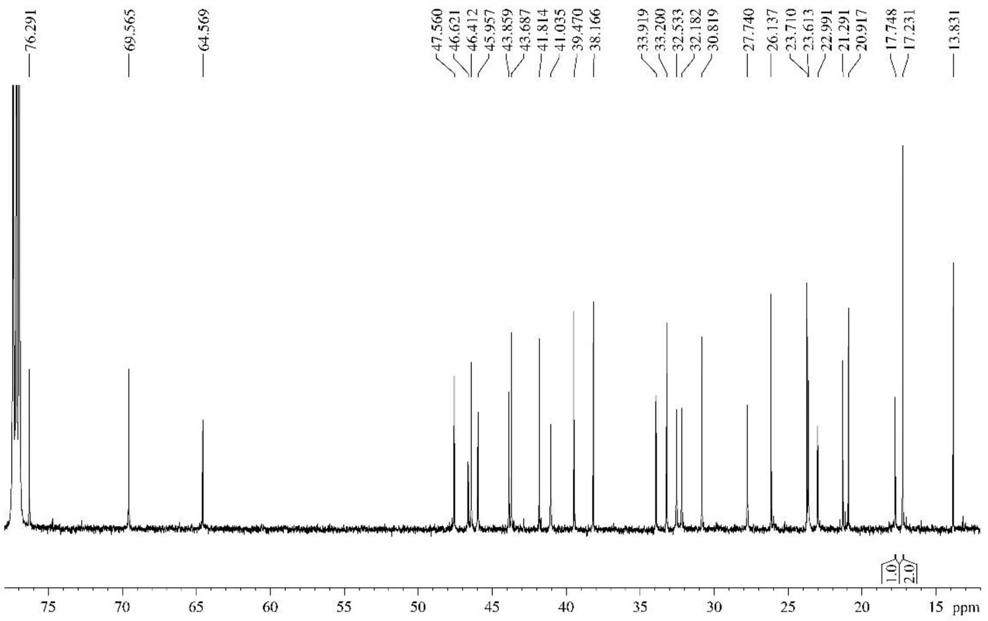

Figure S4. Enlarged ^{13}C NMR (150 MHz, $CDCl_3$) spectrum of compound **1**

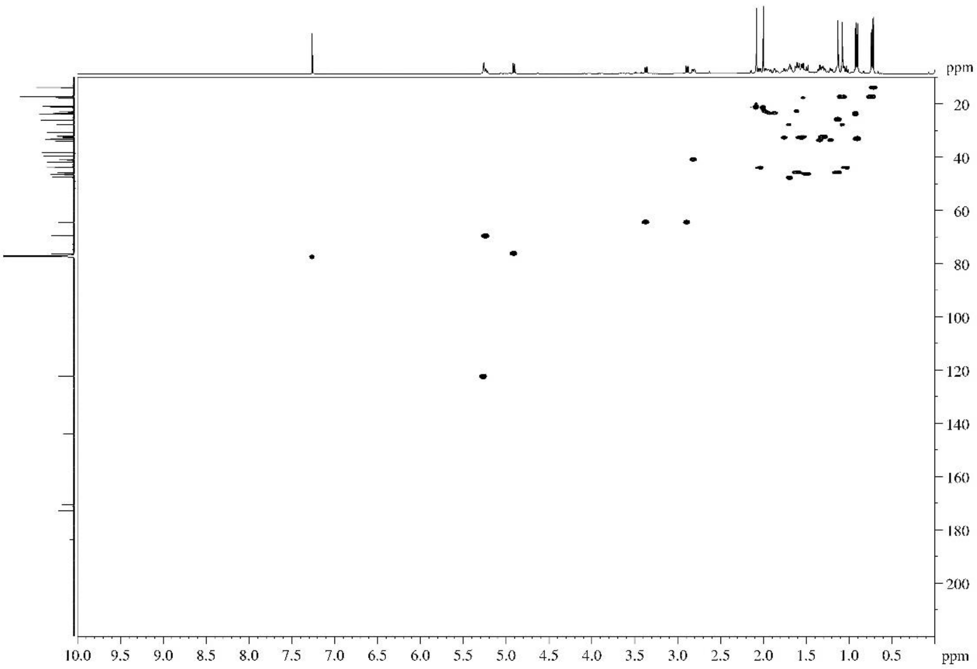

Figure S5. HSQC ($CDCl_3$) spectrum of compound **1**

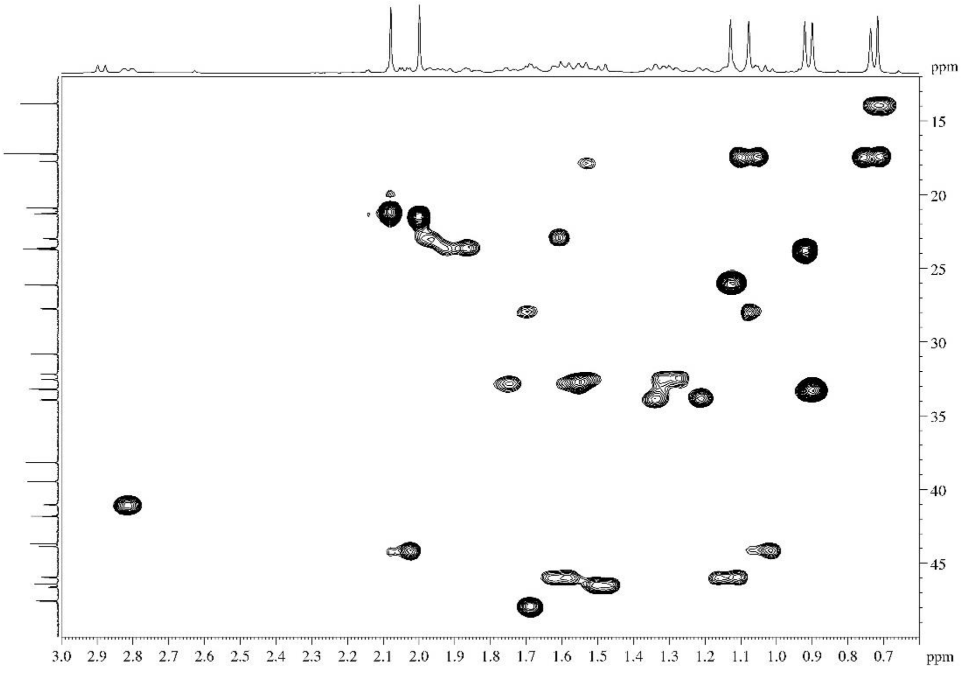

Figure S6. Enlarged HSQC ($CDCl_3$) spectrum of compound **1**

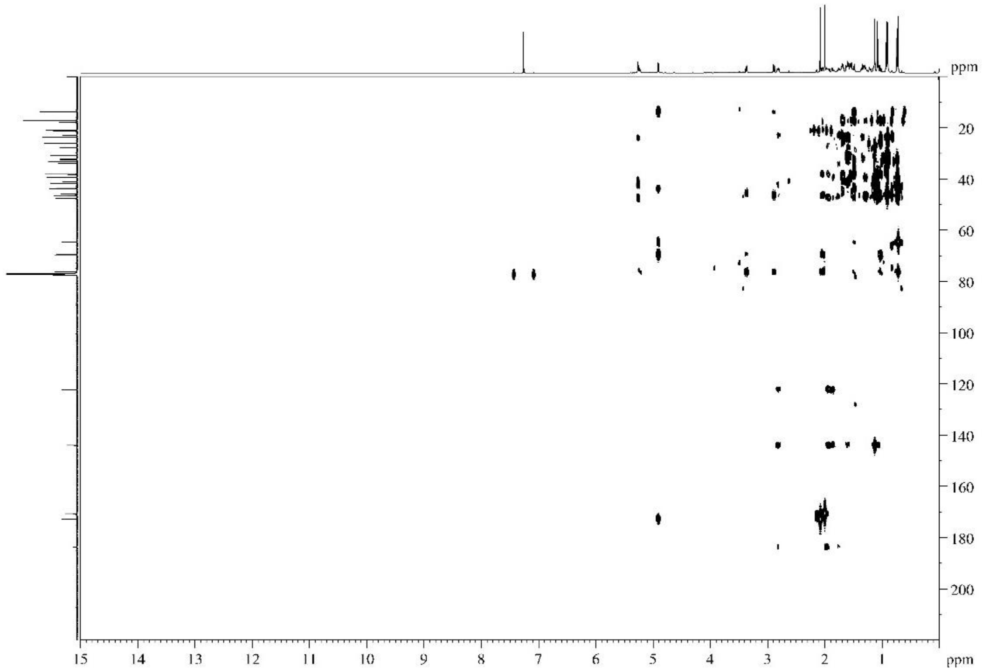

Figure S7. HMBC ($CDCl_3$) spectrum of compound **1**

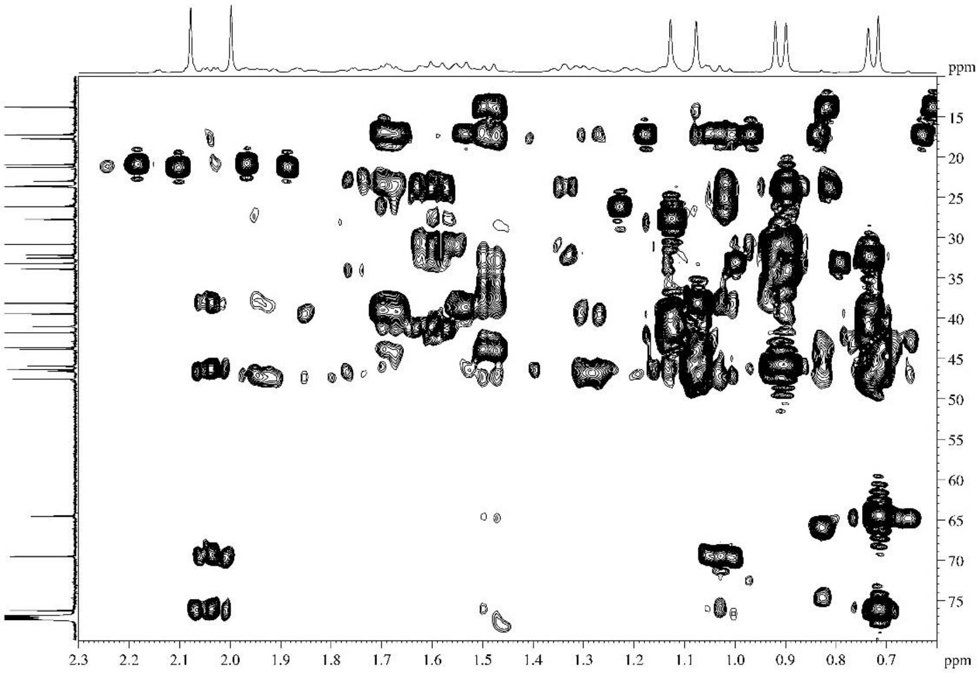

Figure S8. Enlarged HMBC ($CDCl_3$) spectrum of compound **1**

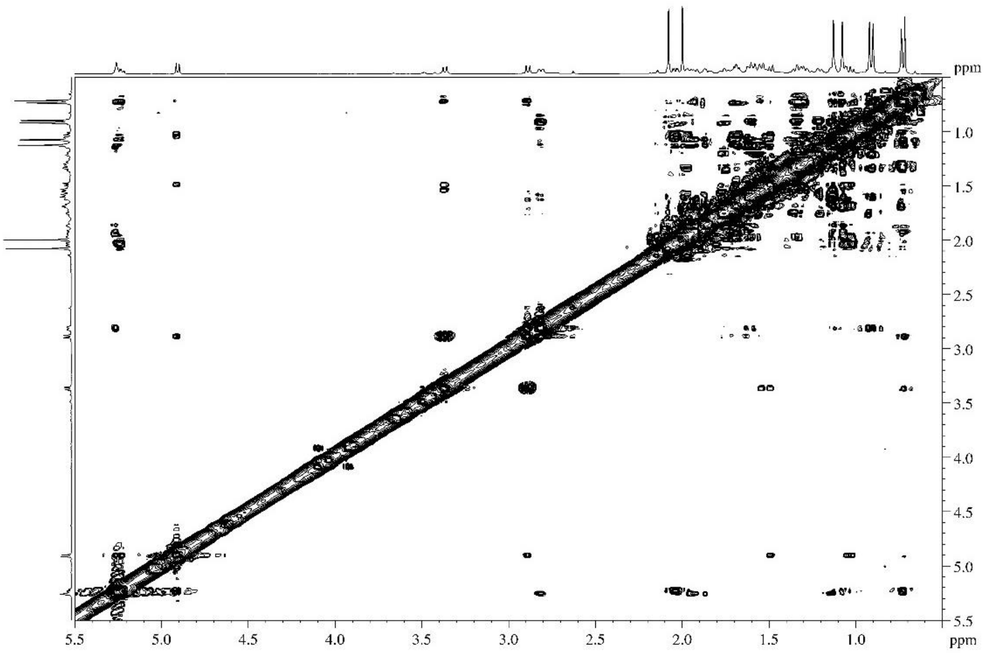

Figure S9. NOESY ($CDCl_3$) spectrum of compound **1**

Qualitative Compound Report

**Data File**	G-930-PXH-4.d	**Sample Name**	G-930-PXH-4
**Sample Type**	Sample	**Position**	P1-A5
**Instrument Name**	Instrument 1	**User Name**	
**Acq Method**	neg-1min.m	**Acquired Time**	10/1/2022 12:33:53 AM
**IRM Calibration Status**	Success	**DA Method**	QG-907.m
**Comment**			
**Sample Group**		**Info.**	
**Stream Name**	LC 1	**Acquisition SW Version**	6200 series TOF/6500 series Q-TOF B.08.00 (B8058.0)

Compound Table

Compound Label	RT	Mass	Abund	Formula	Tgt Mass	Diff (ppm)
Cpd 1: C34 H52 O7	0.126	572.3705	14004	C34 H52 O7	572.3713	-1.38

MS Zoomed Spectrum

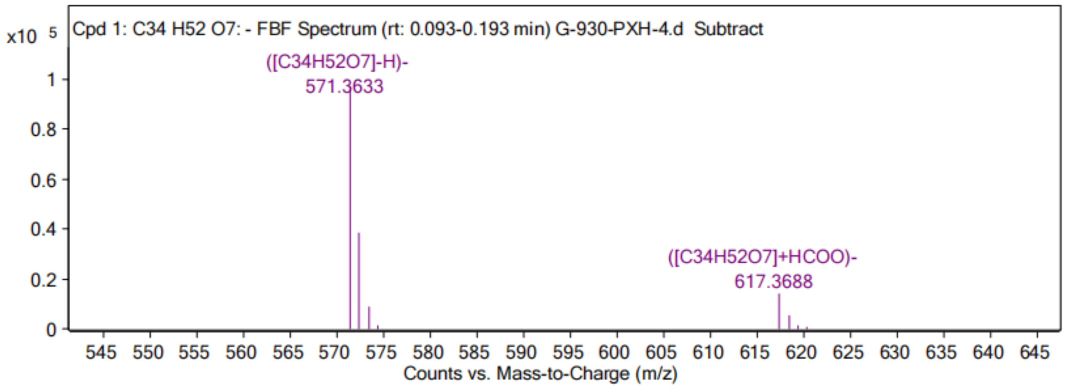

MS Spectrum Peak List

m/z	z	Abund	Formula	Ion
571.3633	1	97269.69	C34H52O7	(M-H)-
572.3666	1	38162.7	C34H52O7	(M-H)-
573.3693	1	8731.45	C34H52O7	(M-H)-
574.3719	1	1331.93	C34H52O7	(M-H)-
617.3688	1	14004.46	C34H52O7	(M+HCOO)-
618.3722	1	5444.76	C34H52O7	(M+HCOO)-
619.3758	1	1369.38	C34H52O7	(M+HCOO)-
620.3795	1	333.19	C34H52O7	(M+HCOO)-

--- End Of Report ---

Figure S10. HRESIMS spectrum of compound **1**

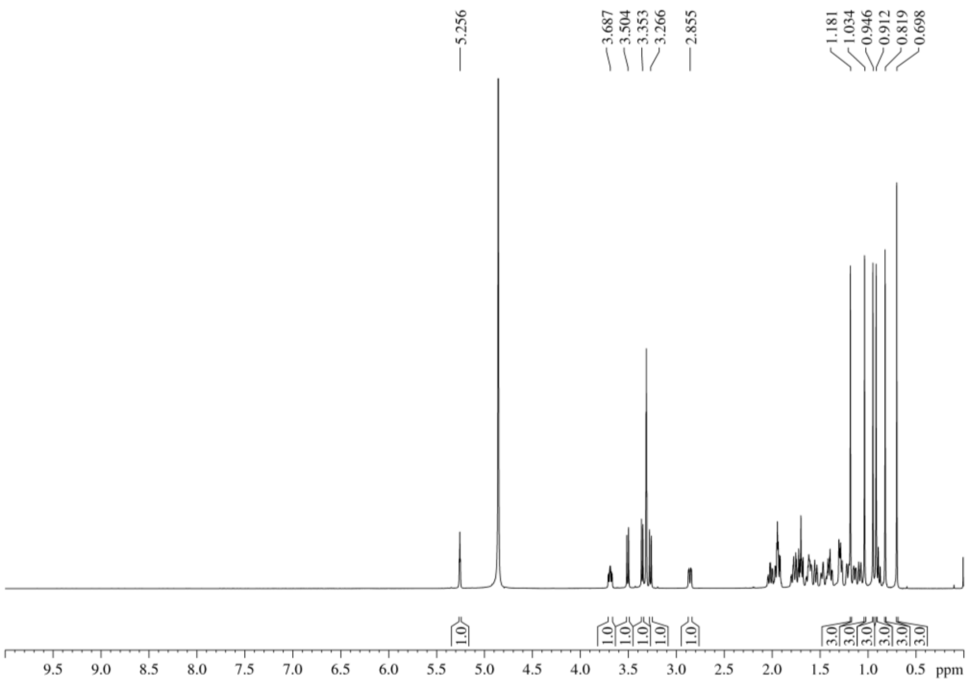

Figure S11. ^{1}H NMR (600 MHz, $CDCl_3$) spectrum of compound **1a**

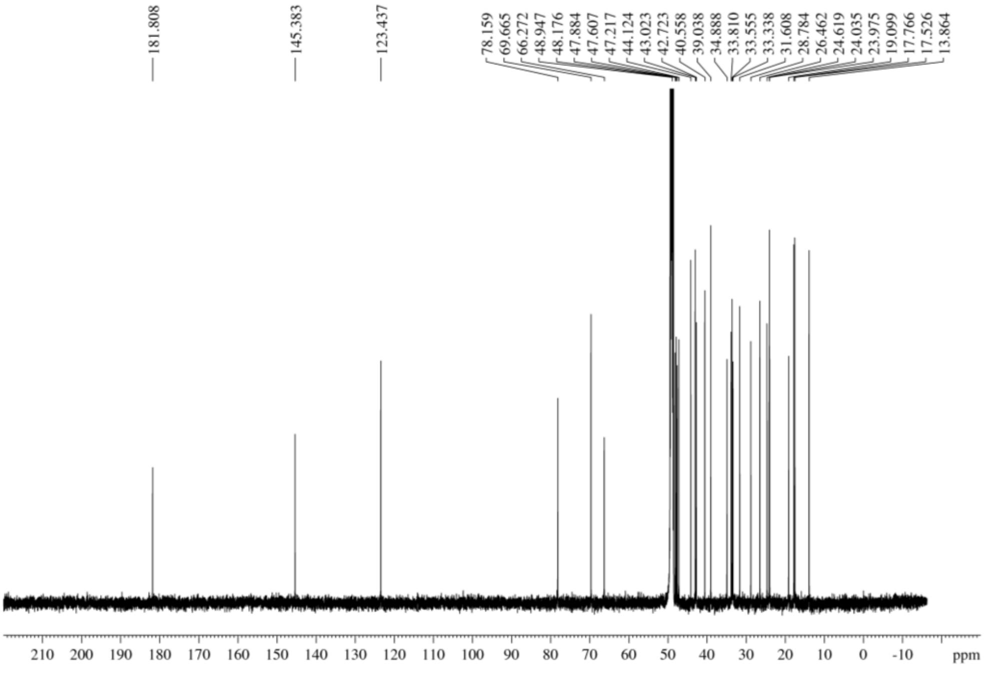

Figure S12. ^{13}C NMR (150 MHz, $CDCl_3$) spectrum of compound **1a**

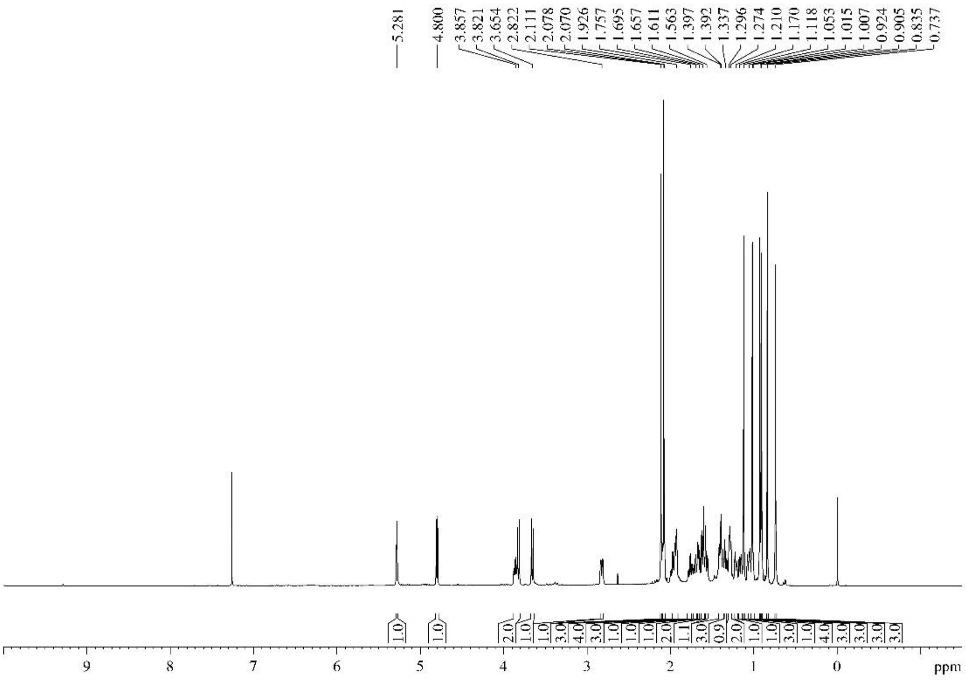

Figure S13. ^1H NMR (600 MHz, $CDCl_3$) spectrum of compound **2**

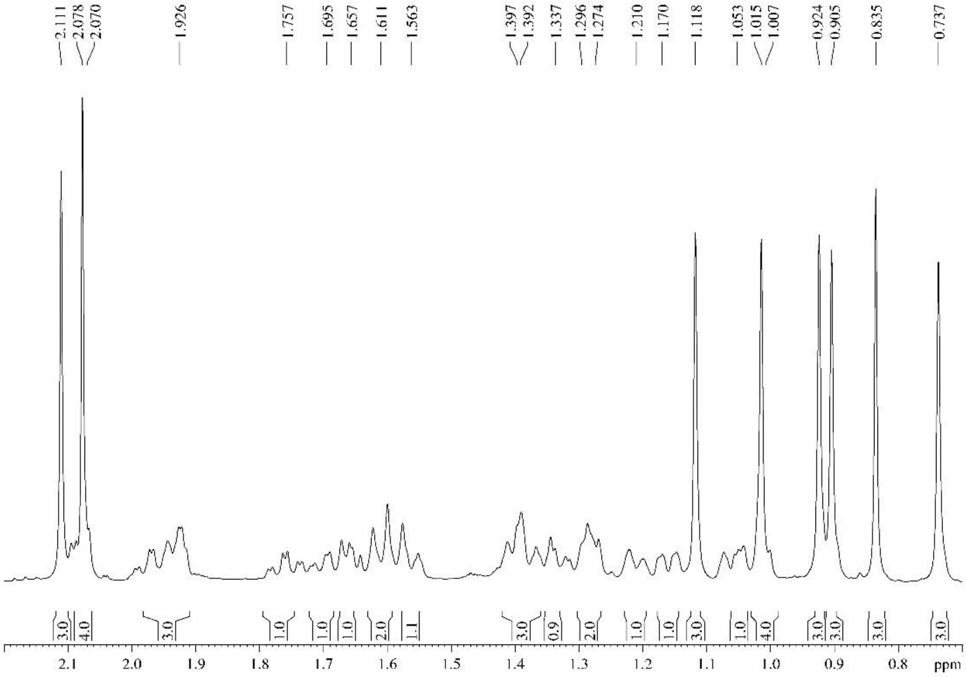

Figure S14. Enlarged ^1H NMR (600 MHz, $CDCl_3$) spectrum of compound **2**

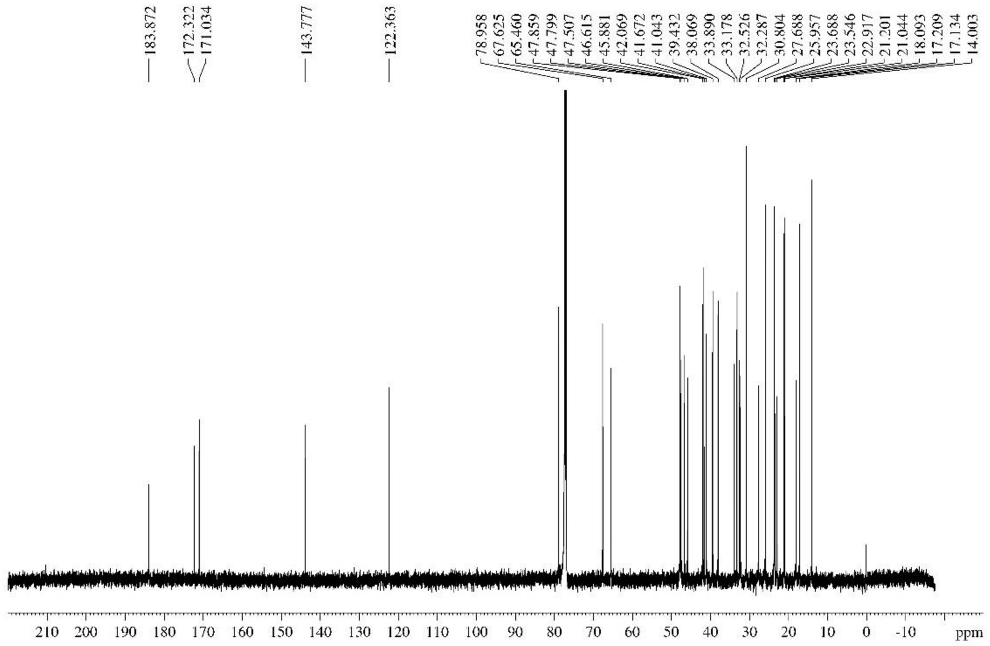

Figure S15. ^{13}C NMR (150 MHz, $CDCl_3$) spectrum of compound **2**

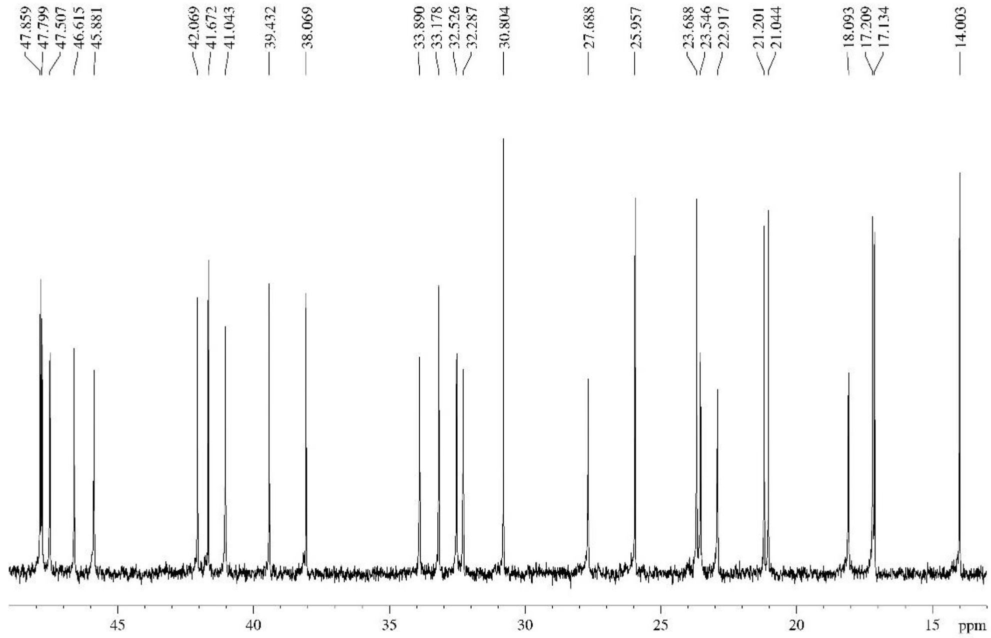

Figure S16. Enlarged ^{13}C NMR (150 MHz, $CDCl_3$) spectrum of compound **2**

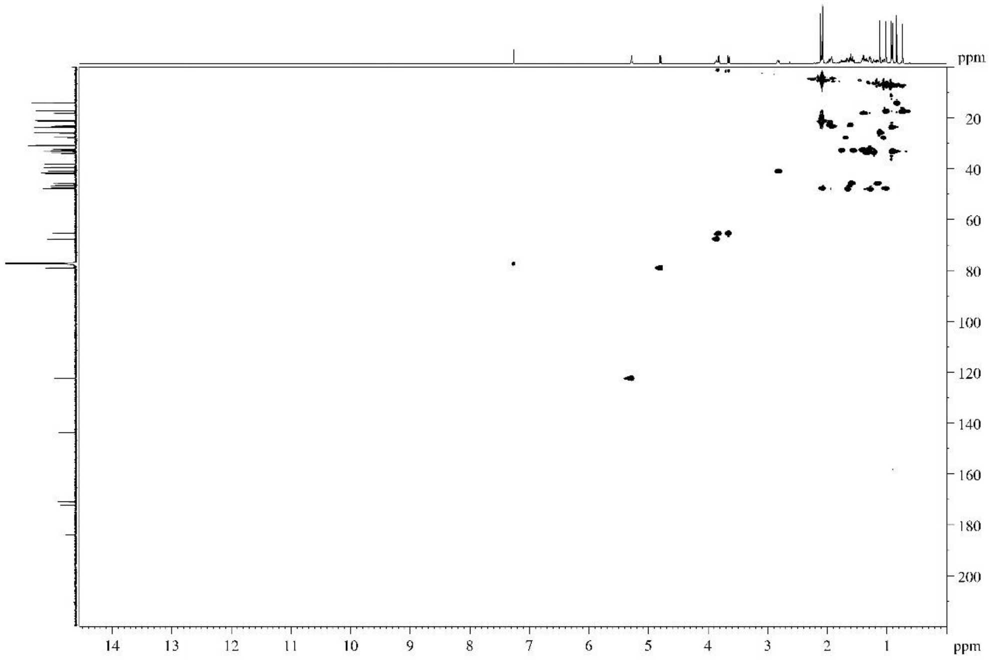

Figure S17. HSQC ($CDCl_3$) spectrum of compound **2**

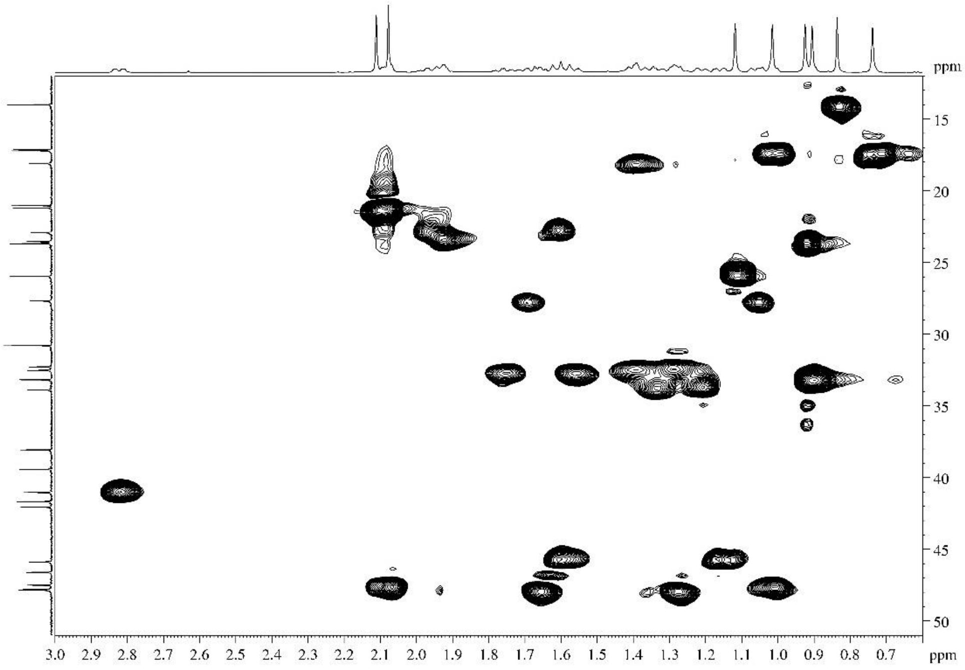

Figure S18. Enlarged HSQC ($CDCl_3$) spectrum of compound **2**

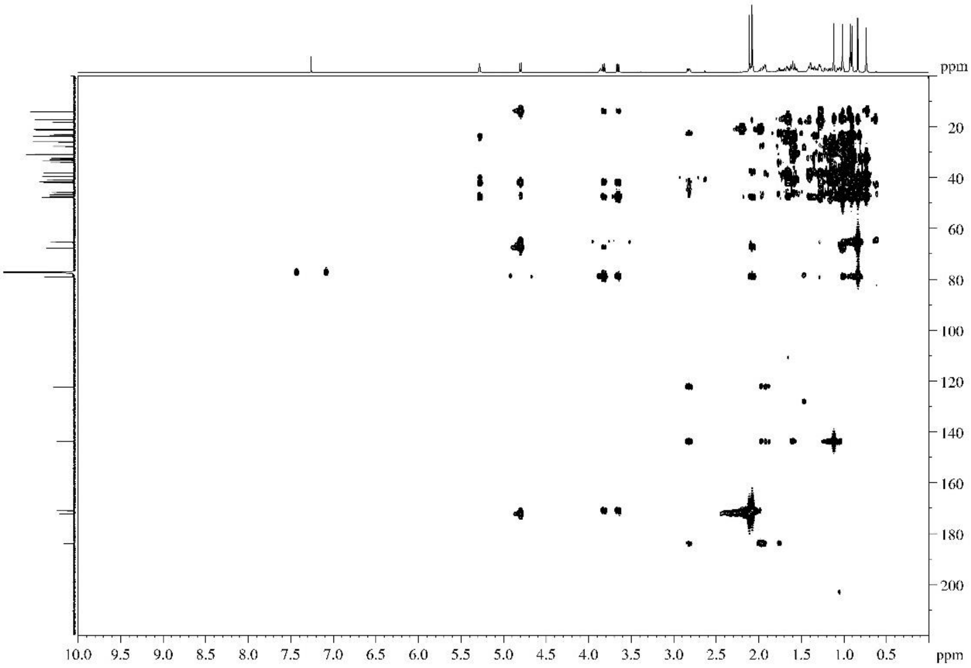

Figure S19. HMBC ($CDCl_3$) spectrum of compound **2**

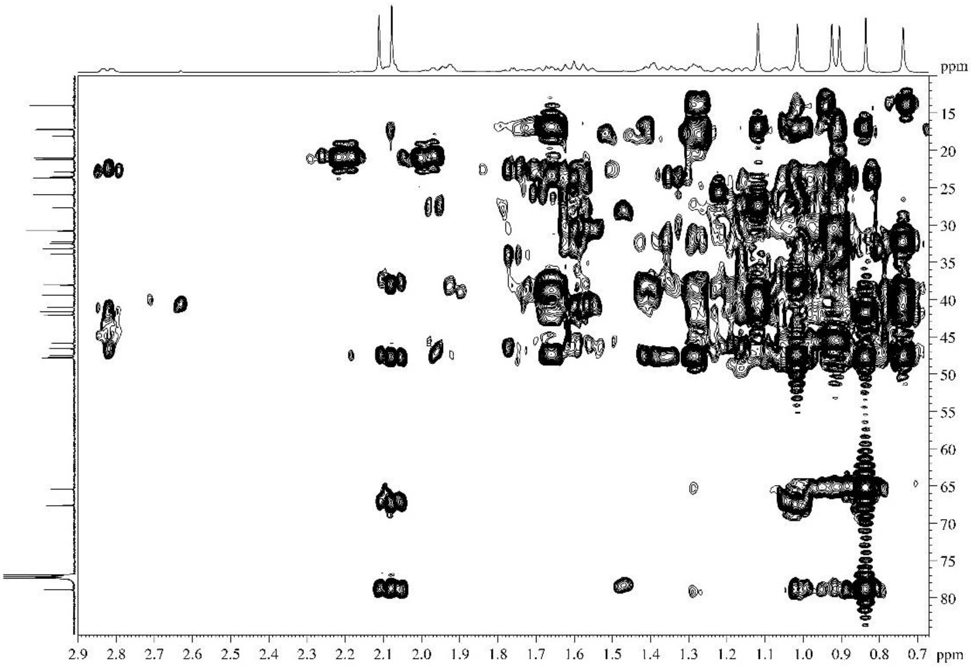

Figure S20. Enlarged HMBC ($CDCl_3$) spectrum of compound **2**

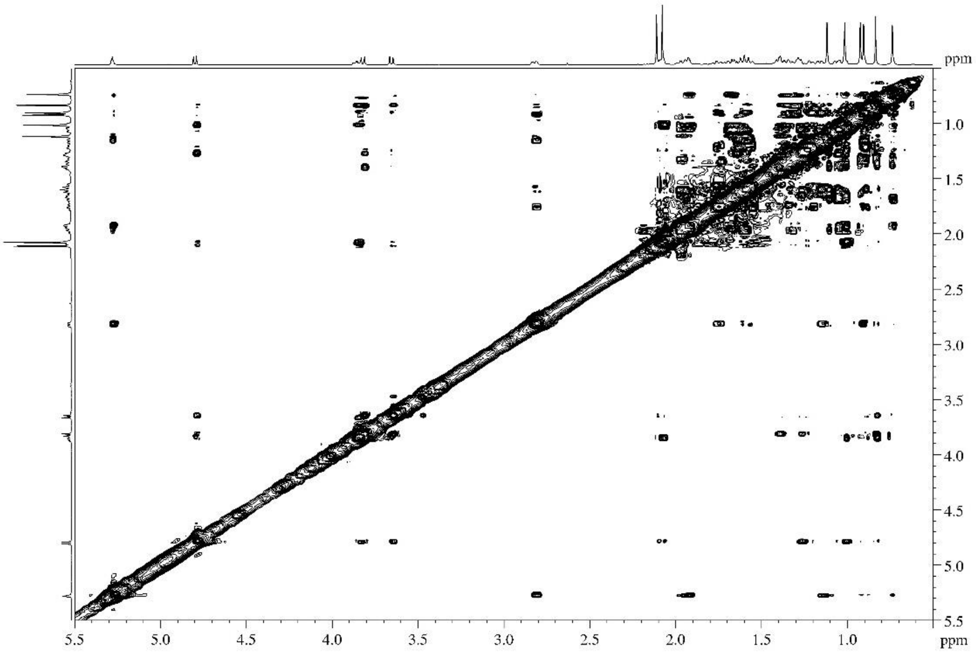

Figure S21. NOESY ($CDCl_3$) spectrum of compound **2**

Qualitative Compound Report

**Data File**	G-930-PXH-3.d	**Sample Name**	G-930-PXH-3
**Sample Type**	Sample	**Position**	P1-A4
**Instrument Name**	Instrument 1	**User Name**	
**Acq Method**	neg-1min.m	**Acquired Time**	10/1/2022 12:32:00 AM
**IRM Calibration Status**	Success	**DA Method**	QG-907.m
**Comment**			
**Sample Group**		**Info.**	
**Stream Name**	LC 1	**Acquisition SW Version**	6200 series TOF/6500 series Q-TOF B.08.00 (B8058.0)

Compound Table

Compound Label	RT	Mass	Abund	Formula	Tgt Mass	Diff (ppm)
Cpd 1: C34 H52 O7	0.123	572.3711	623414	C34 H52 O7	572.3713	-0.35

MS Zoomed Spectrum

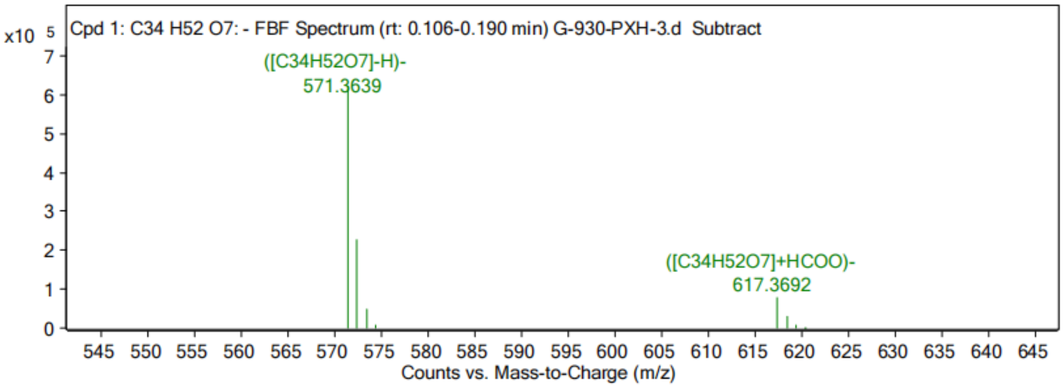

MS Spectrum Peak List

m/z	z	Abund	Formula	Ion
571.3639	1	623414.31	C34H52O7	(M-H)-
572.3673	1	225592.55	C34H52O7	(M-H)-
573.3699	1	49404.84	C34H52O7	(M-H)-
574.372	1	7783.08	C34H52O7	(M-H)-
617.3692	1	78249.3	C34H52O7	(M+HCOO)-
618.3725	1	31253.46	C34H52O7	(M+HCOO)-
619.3758	1	7448.57	C34H52O7	(M+HCOO)-
620.3779	1	1516.8	C34H52O7	(M+HCOO)-

--- End Of Report ---

Figure S22. HRESIMS spectrum of compound **2**

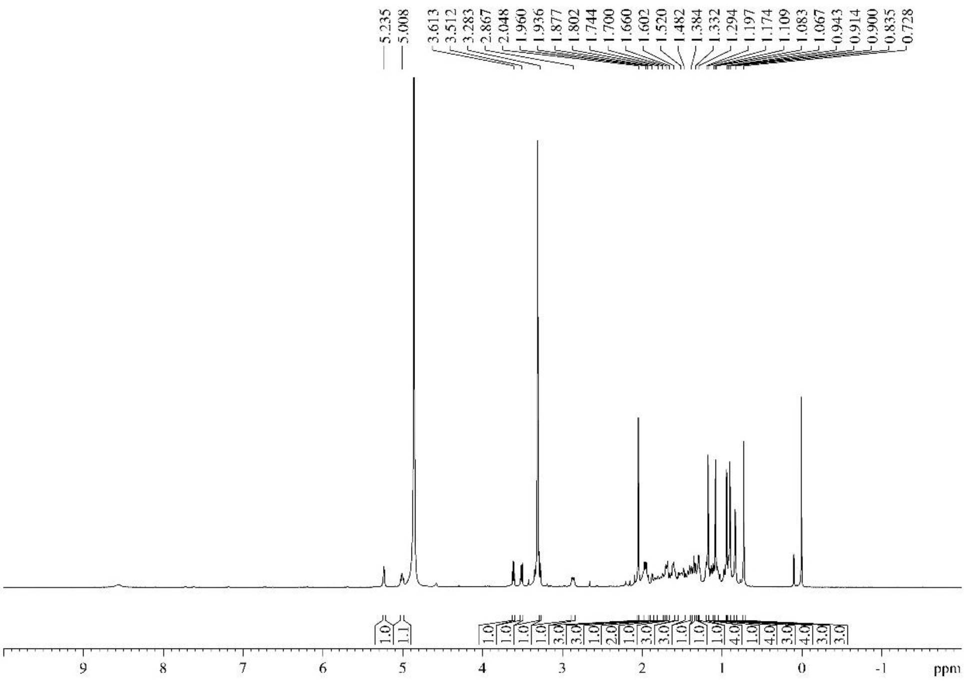

Figure S23. ^{1}H NMR (600 MHz, CD_3OD) spectrum of compound **3**

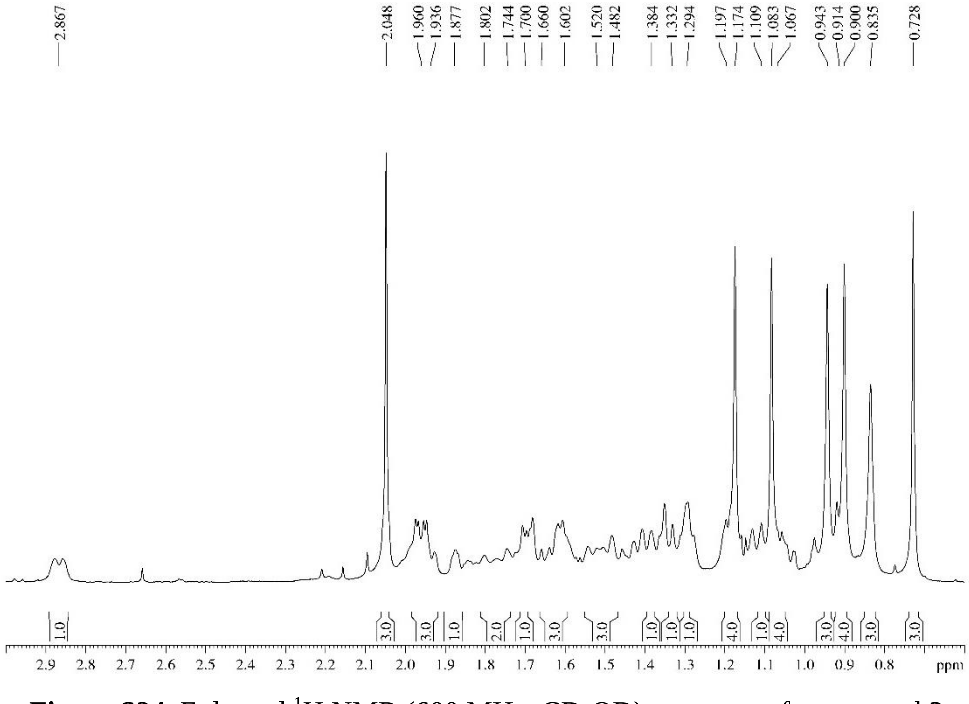

Figure S24. Enlarged ^{1}H NMR (600 MHz, CD_3OD) spectrum of compound **3**

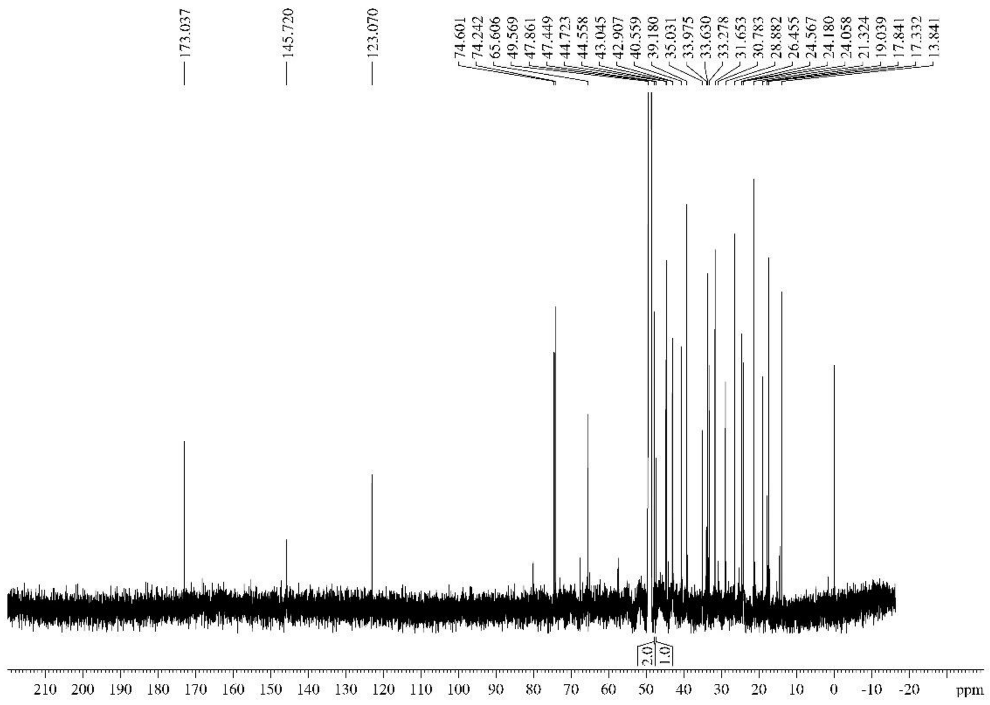

Figure S25. ^{13}C NMR (150 MHz, CD_3OD) spectrum of compound **3**

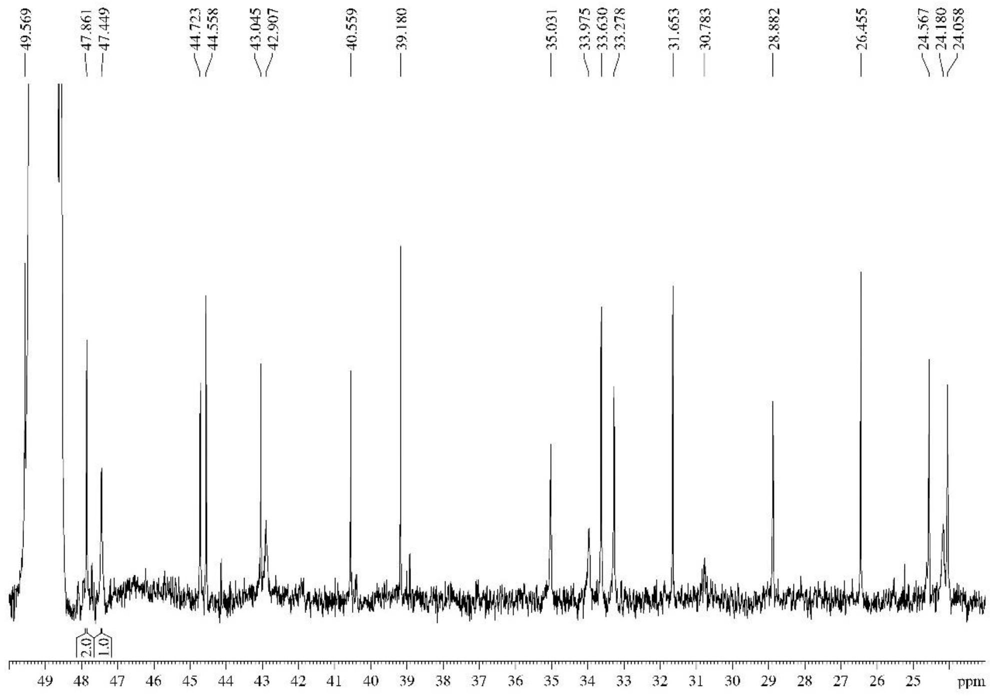

Figure S26. Enlarged ^{13}C NMR (150 MHz, CD_3OD) spectrum of compound **3**

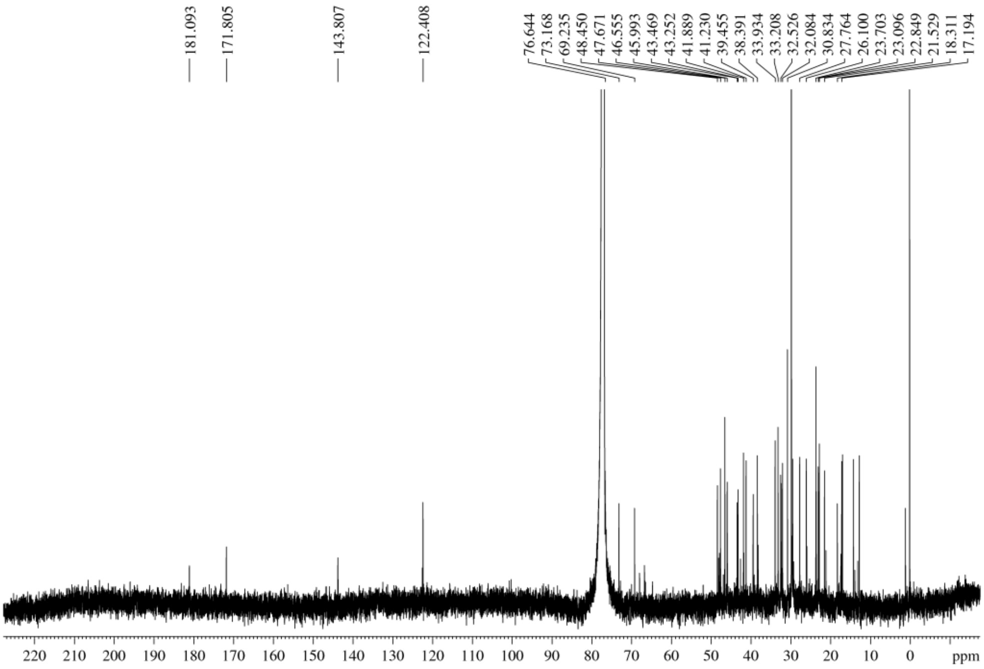

Figure S27. ^{13}C NMR (150 MHz, CD_3Cl) spectrum of compound **3**

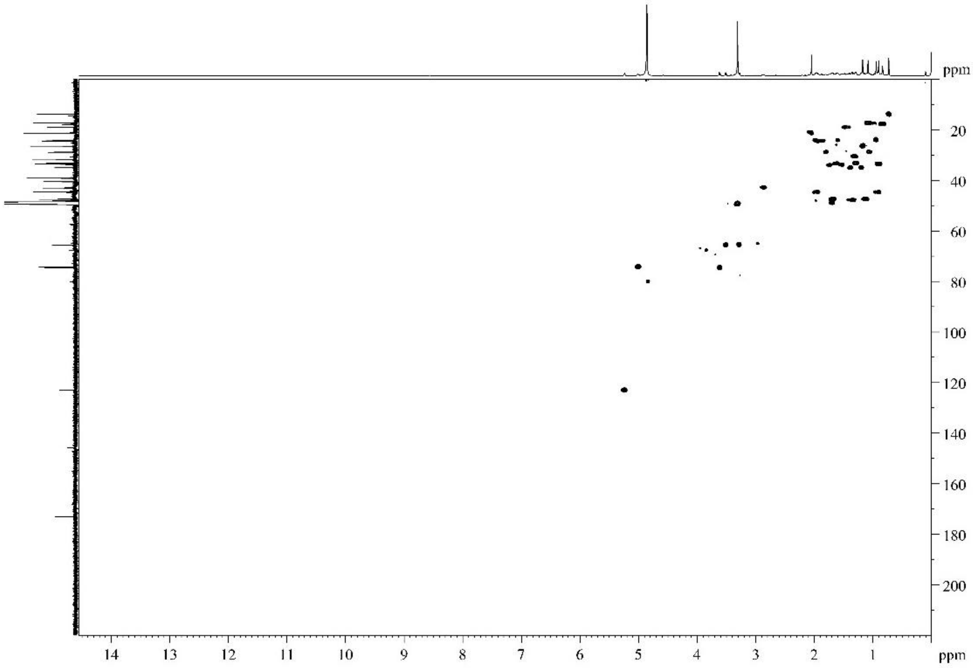

Figure S28. HSQC (CD_3OD) spectrum of compound **3**

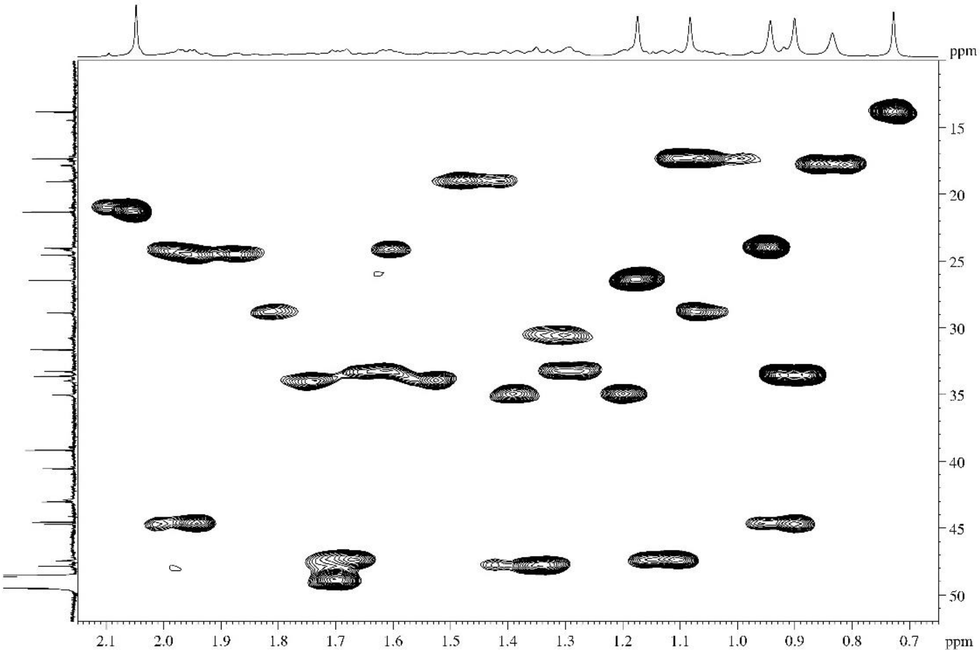

Figure S29. Enlarged HSQC (CD_3OD) spectrum of compound **3**

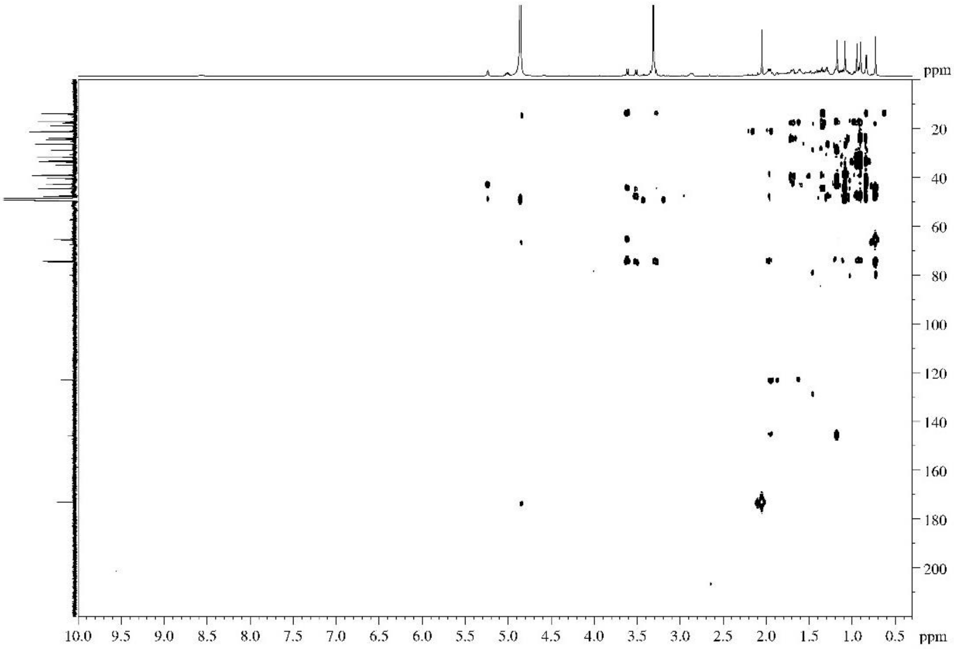

Figure S30. HMBC (CD_3OD) spectrum of compound **3**

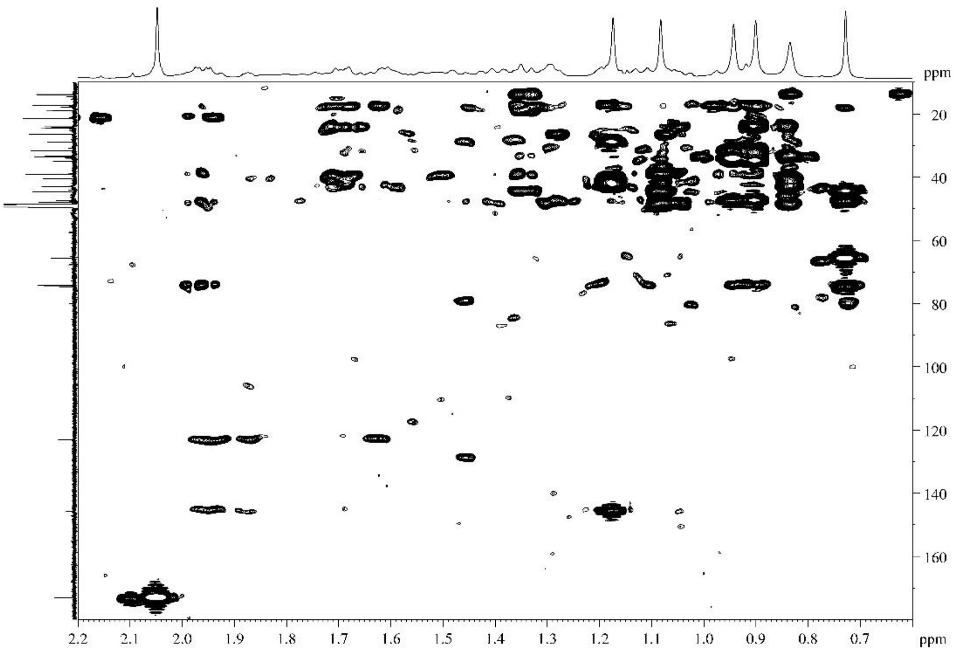

Figure S31. Enlarged HMBC (CD_3OD) spectrum of compound **3**

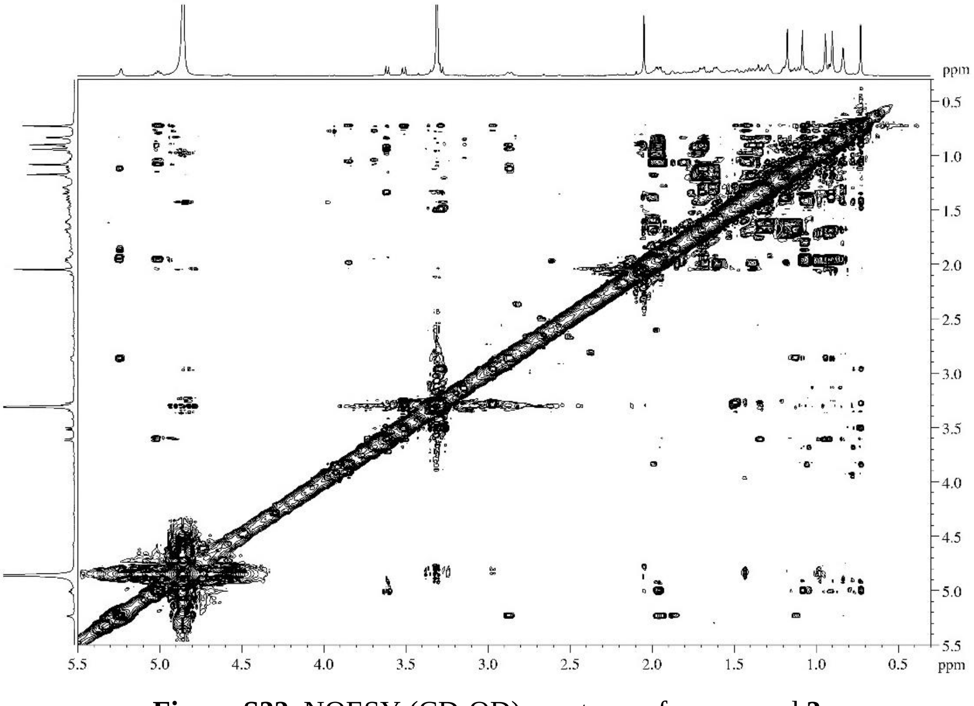

Figure S32. NOESY (CD_3OD) spectrum of compound **3**

Qualitative Compound Report

**Data File**	G-1013-PXH-2.d	**Sample Name**	G-1013-PXH-2
**Sample Type**	Sample	**Position**	P1-A2
**Instrument Name**	Instrument 1	**User Name**	
**Acq Method**	pos-1min.m	**Acquired Time**	10/13/2022 9:56:52 AM
**IRM Calibration Status**	Success	**DA Method**	QG-907.m
**Comment**			
**Sample Group**		**Info.**	
**Stream Name**	LC 1	**Acquisition SW Version**	6200 series TOF/6500 series Q-TOF B.08.00 (B8058.0)

Compound Table

Compound Label	RT	Mass	Abund	Formula	Tgt Mass	Diff (ppm)
Cpd 1: C32 H50 O6	0.115	530.3621	20647	C32 H50 O6	530.3607	2.63

MS Zoomed Spectrum

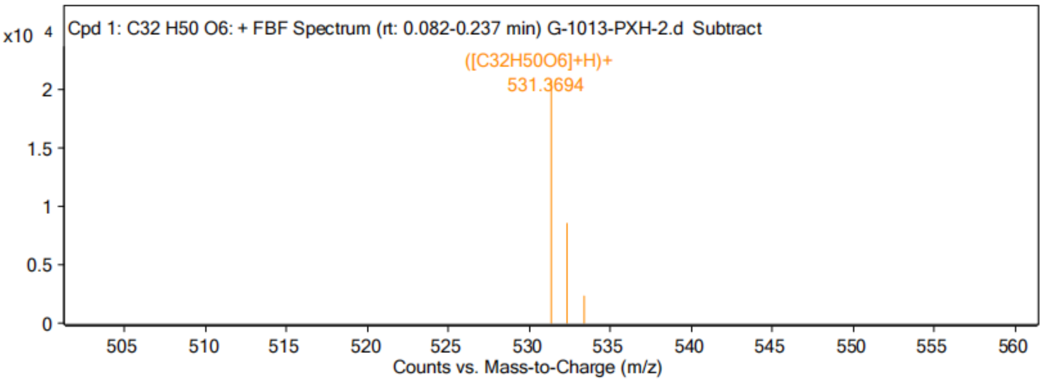

MS Spectrum Peak List

m/z	z	Abund	Formula	Ion
531.3694	1	20646.85	C32H50O6	(M+H)+
532.3737	1	8576.44	C32H50O6	(M+H)+
533.3729	1	2347.85	C32H50O6	(M+H)+

--- End Of Report ---

Figure S33. HRESIMS spectrum of compound **3**

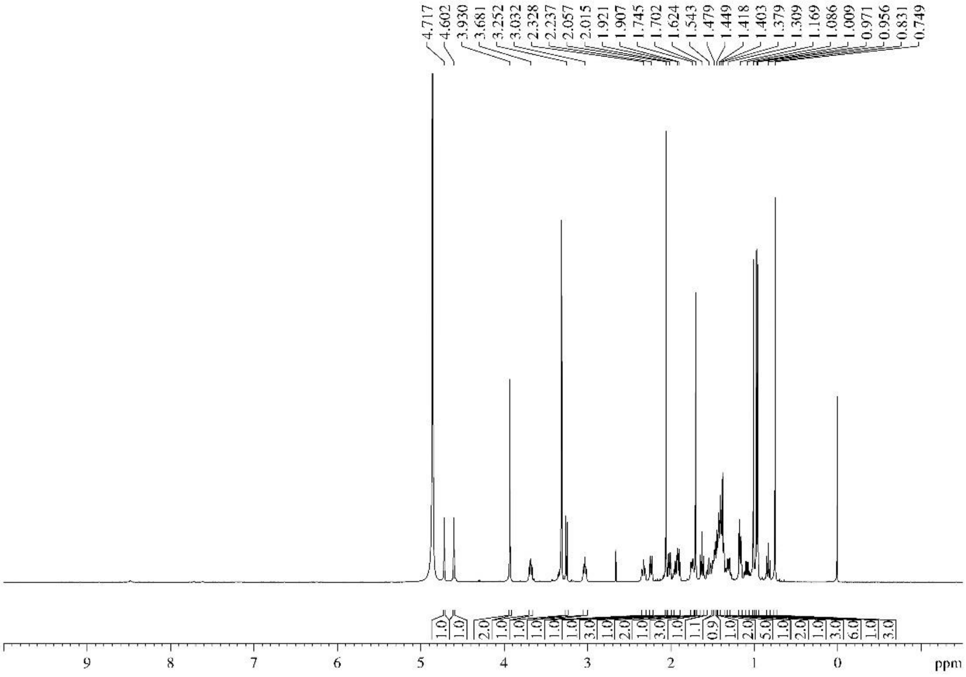

Figure S34. ^{1}H NMR (600 MHz, CD_3OD) spectrum of compound **4**

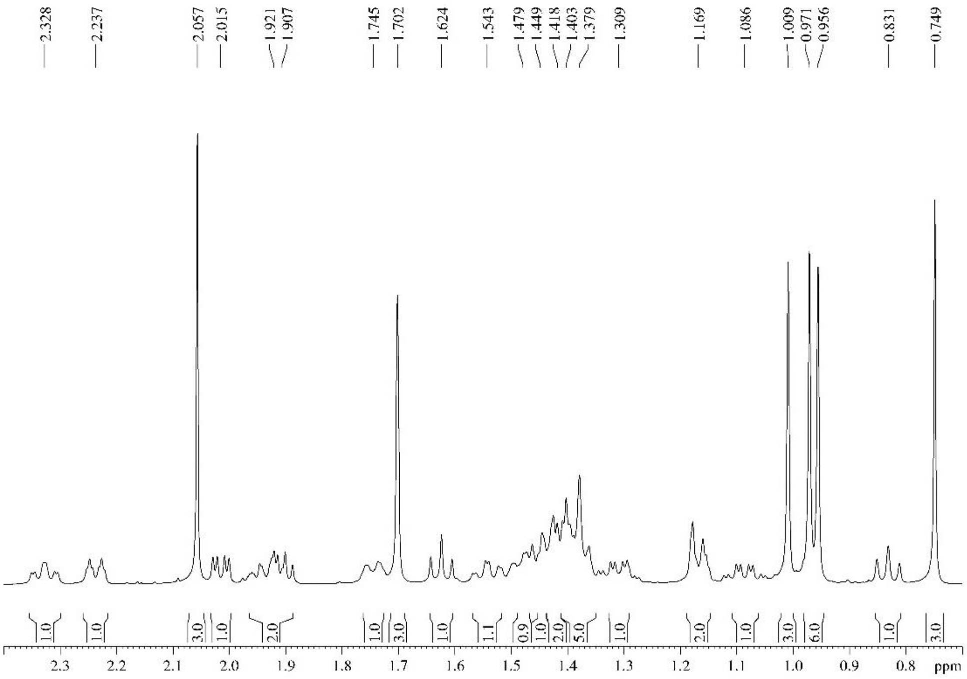

Figure S35. Enlarged ^{1}H NMR (600 MHz, CD_3OD) spectrum of compound **4**

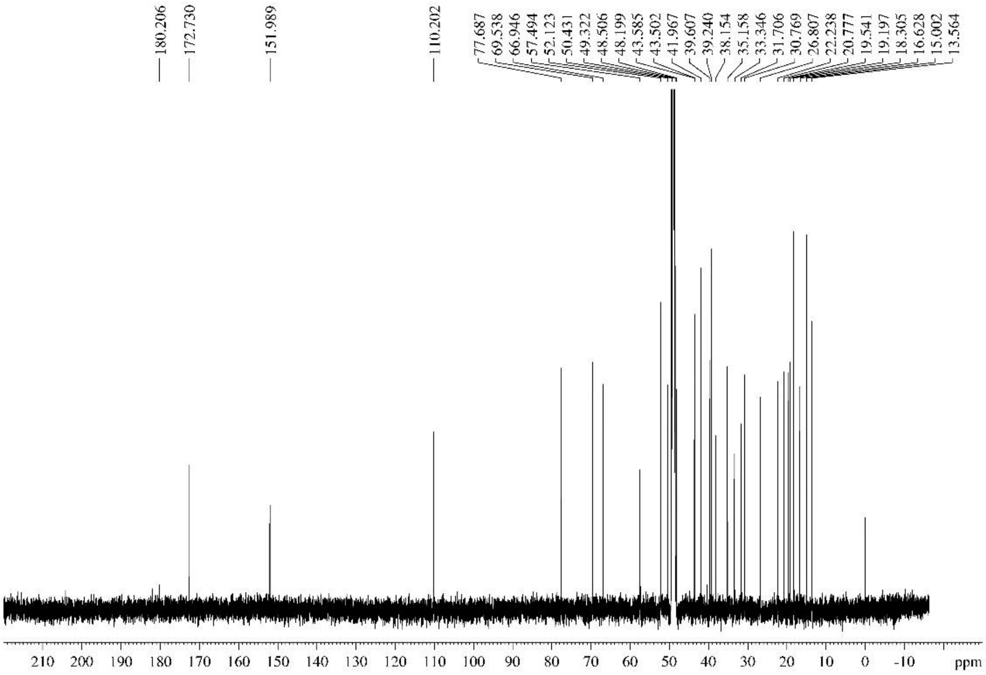

Figure S36. ^{13}C NMR (150 MHz, CD_3OD) spectrum of compound **4**

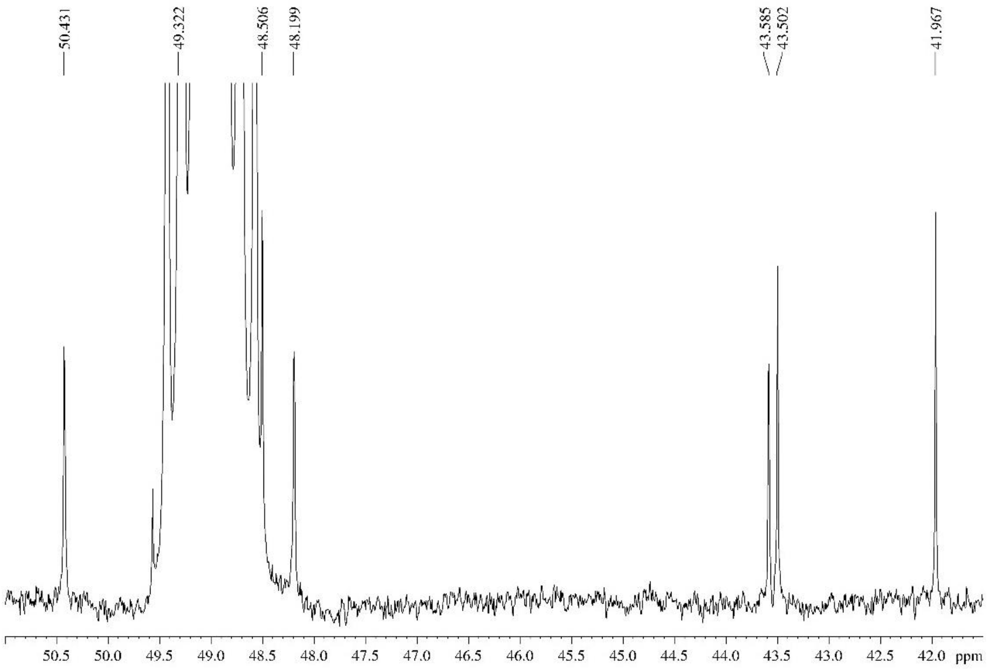

Figure S37. Enlarged ^{13}C NMR (150 MHz, CD_3OD) spectrum of compound **4**

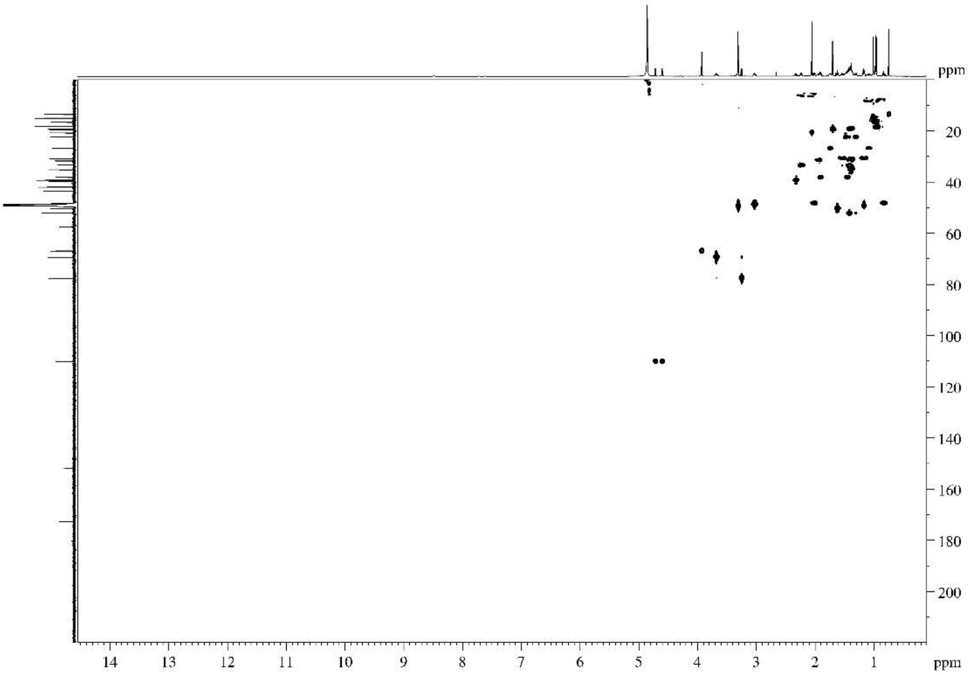

Figure S38. HSQC (CD_3OD) spectrum of compound **4**

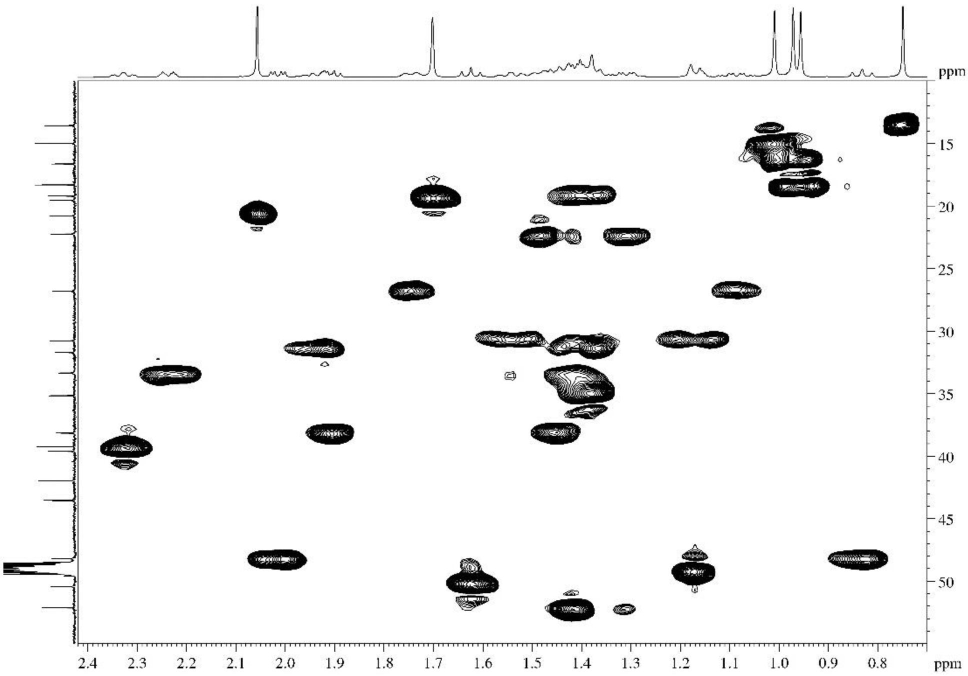

Figure S39. Enlarged HSQC (CD_3OD) spectrum of compound **4**

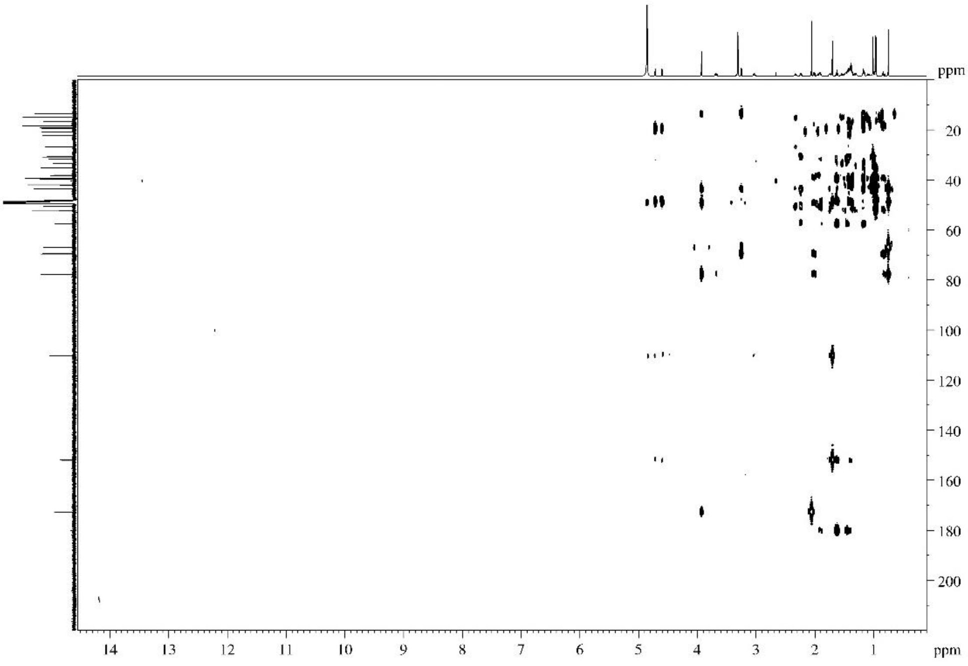

Figure S40. HMBC (CD_3OD) spectrum of compound **4**

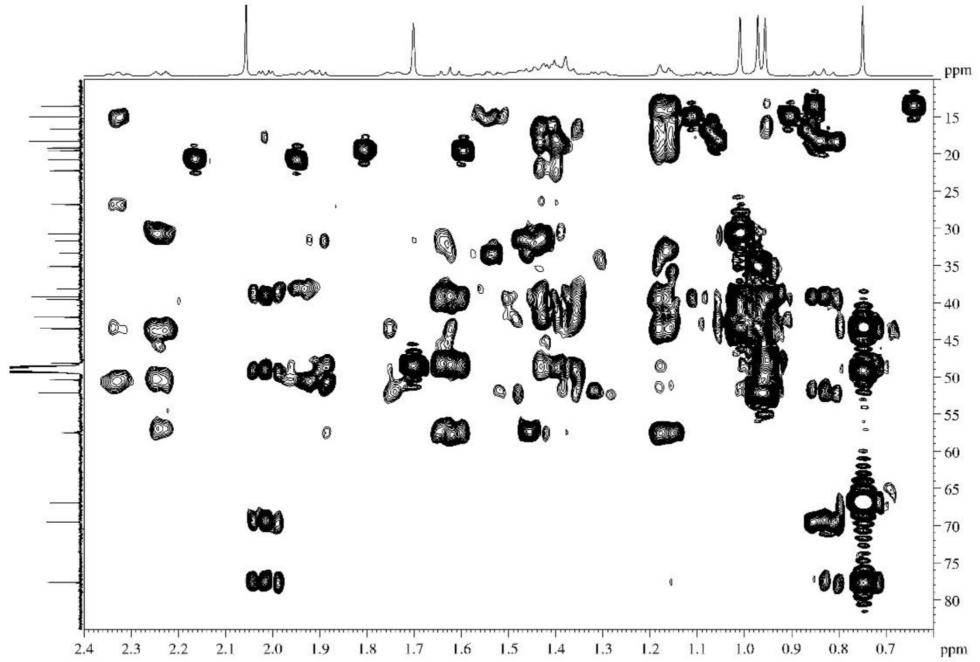

Figure S41. Enlarged HMBC (CD_3OD) spectrum of compound **4**

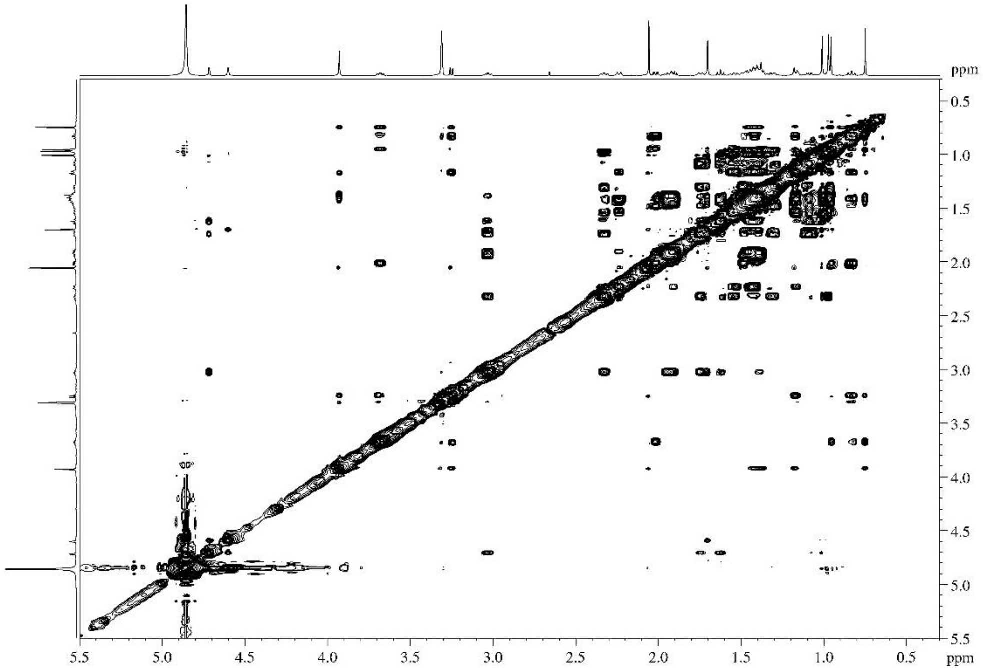

Figure S42. NOESY (CD_3OD) spectrum of compound **4**

Qualitative Compound Report

**Data File**	G-930-PXH-1.d	**Sample Name**	G-930-PXH-1
**Sample Type**	Sample	**Position**	P1-A2
**Instrument Name**	Instrument 1	**User Name**	
**Acq Method**	neg-1min.m	**Acquired Time**	10/1/2022 12:28:14 AM
**IRM Calibration Status**	Success	**DA Method**	QG-907.m
**Comment**			
**Sample Group**		**Info.**	
**Stream Name**	LC 1	**Acquisition SW Version**	6200 series TOF/6500 series Q-TOF B.08.00 (B8058.0)

Compound Table

Compound Label	RT	Mass	Abund	Formula	Tgt Mass	Diff (ppm)
Cpd 1: C32 H50 O6	0.132	530.36	212429	C32 H50 O6	530.3607	-1.32

MS Zoomed Spectrum

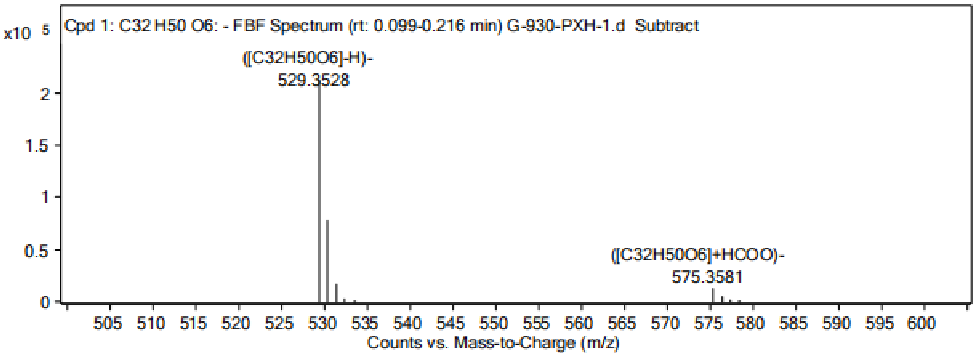

MS Spectrum Peak List

m/z	z	Abund	Formula	Ion
529.3528	1	212428.7	C32H50O6	(M-H)-
530.3562	1	77758.04	C32H50O6	(M-H)-
531.3587	1	16262.82	C32H50O6	(M-H)-
532.3616	1	2581.88	C32H50O6	(M-H)-
533.3621	1	310.57	C32H50O6	(M-H)-
575.3581	1	12352.63	C32H50O6	(M+HCOO)-
576.3616	1	4563.06	C32H50O6	(M+HCOO)-
577.3655	1	1193.37	C32H50O6	(M+HCOO)-
578.3648	1	213.25	C32H50O6	(M+HCOO)-

--- End Of Report ---

Figure S43. HRESIMS spectrum of compound **4**

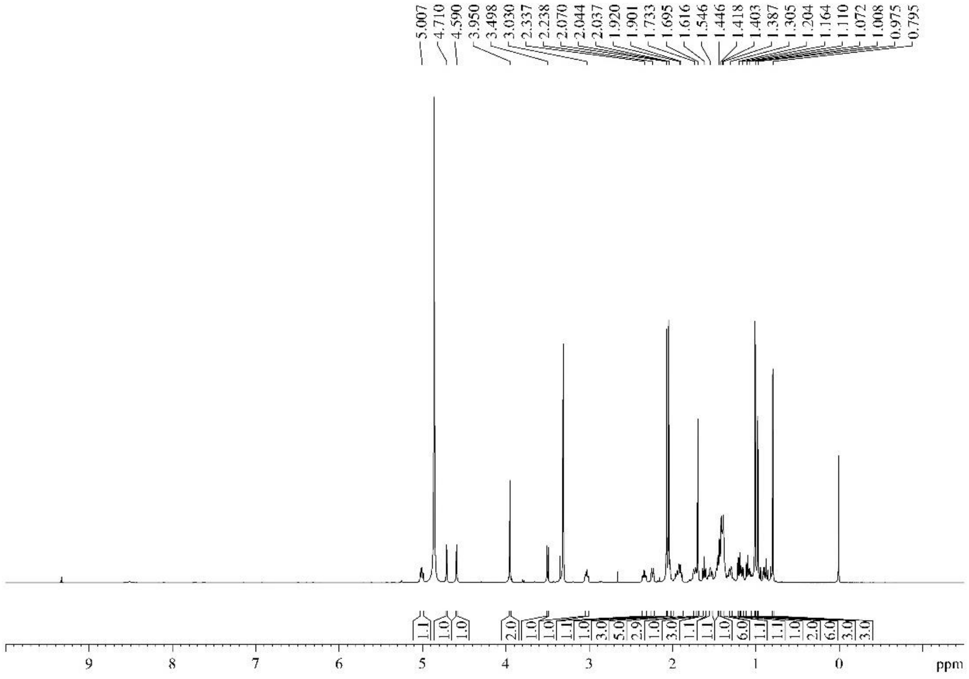

Figure S44. ^{1}H NMR (600 MHz, CD_3OD) spectrum of compound **5**

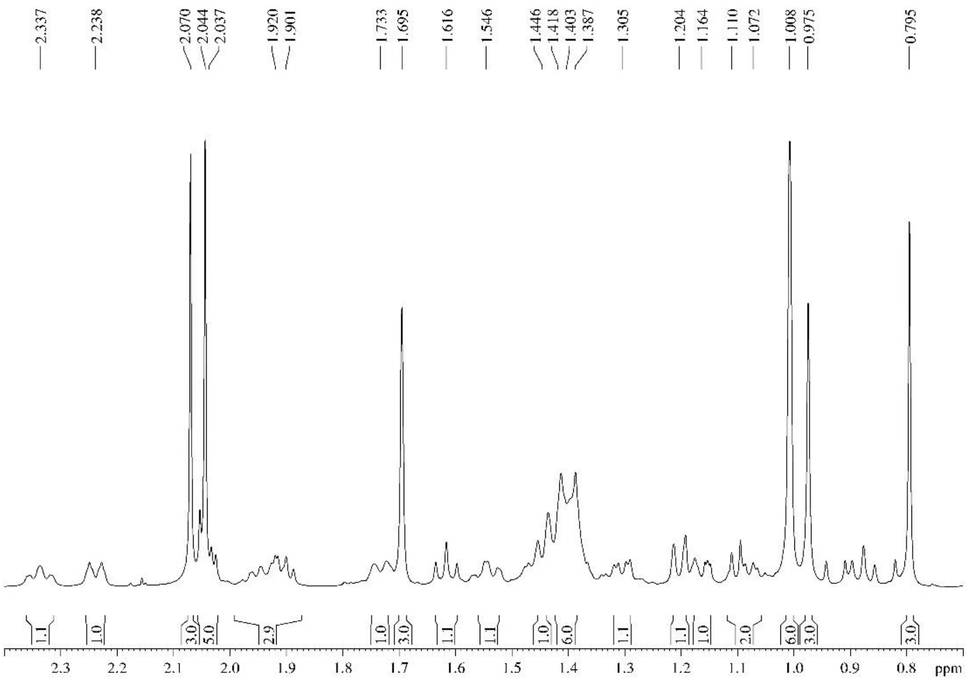

Figure S45. Enlarged ^{1}H NMR (600 MHz, CD_3OD) spectrum of compound **5**

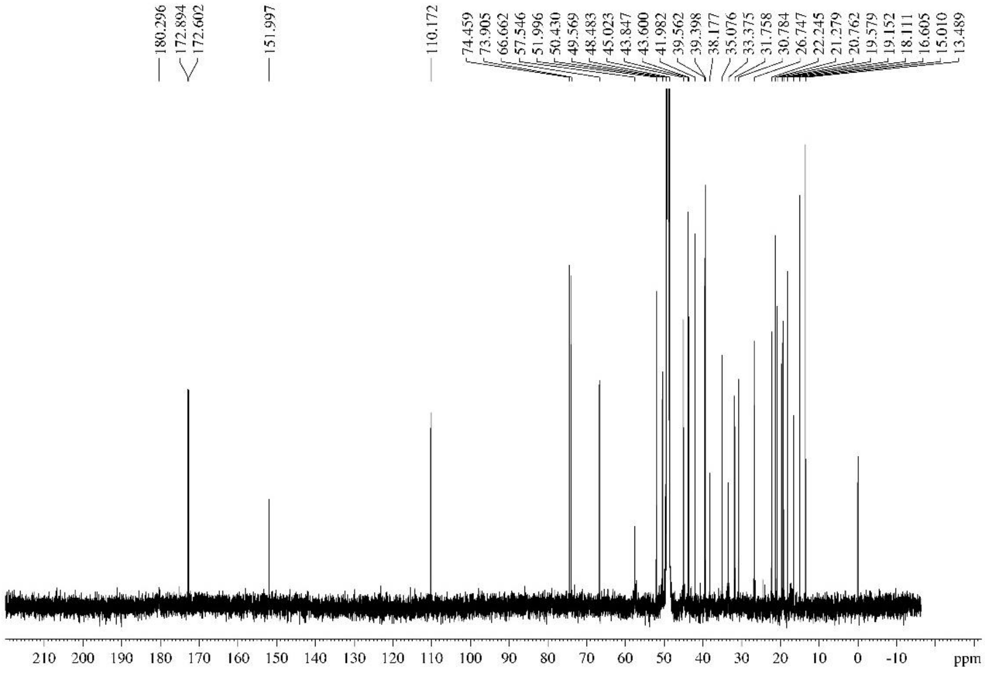

Figure S46. ^{13}C NMR (150 MHz, CD_3OD) spectrum of compound **5**

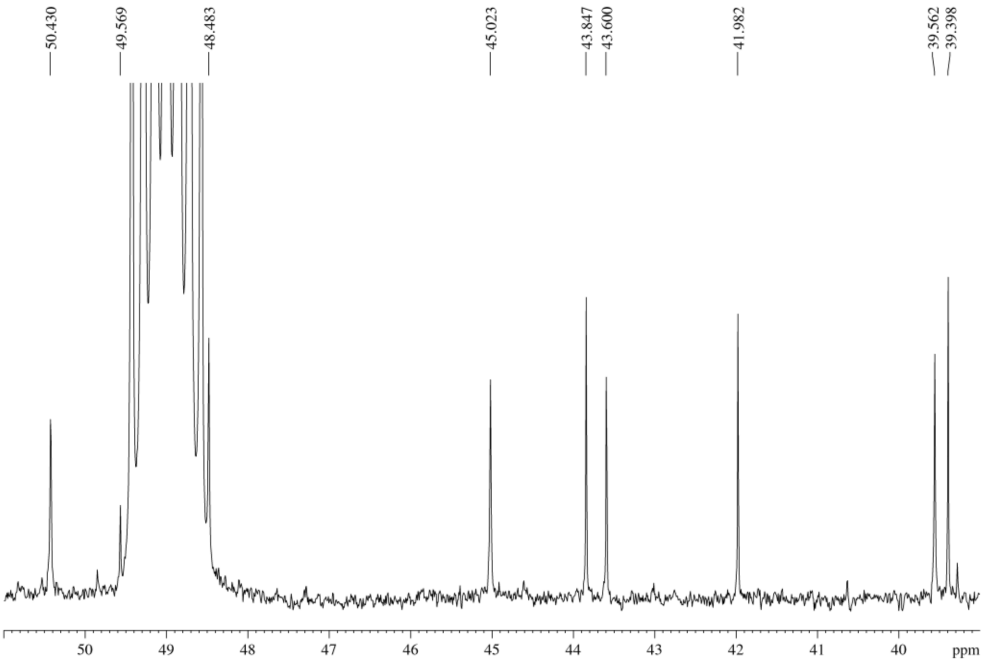

Figure S47. Enlarged ^{13}C NMR (150 MHz, CD_3OD) spectrum of compound **5**

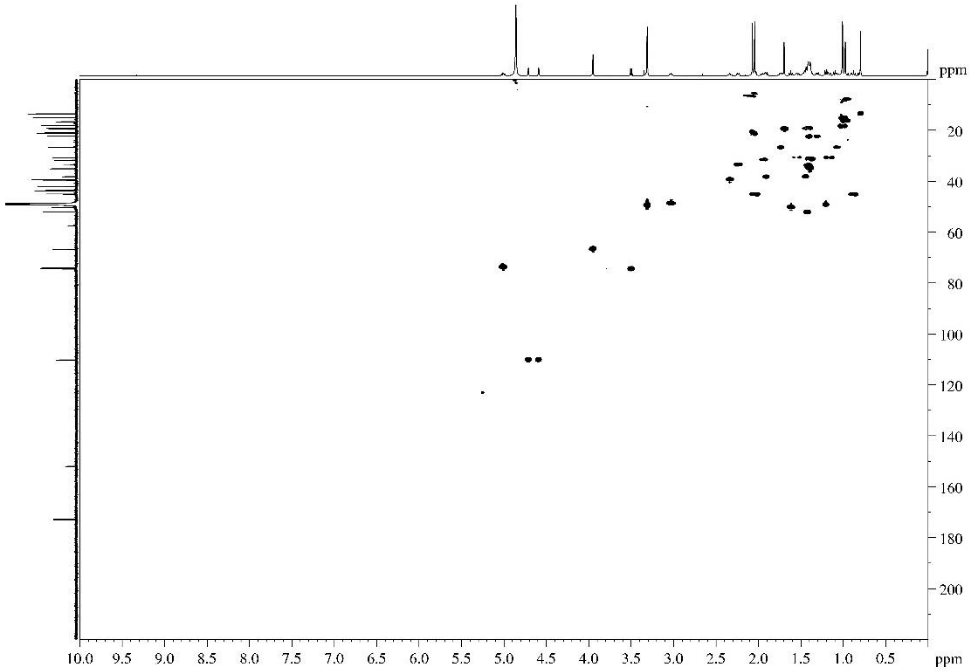

Figure S48. HSQC (CD_3OD) spectrum of compound **5**

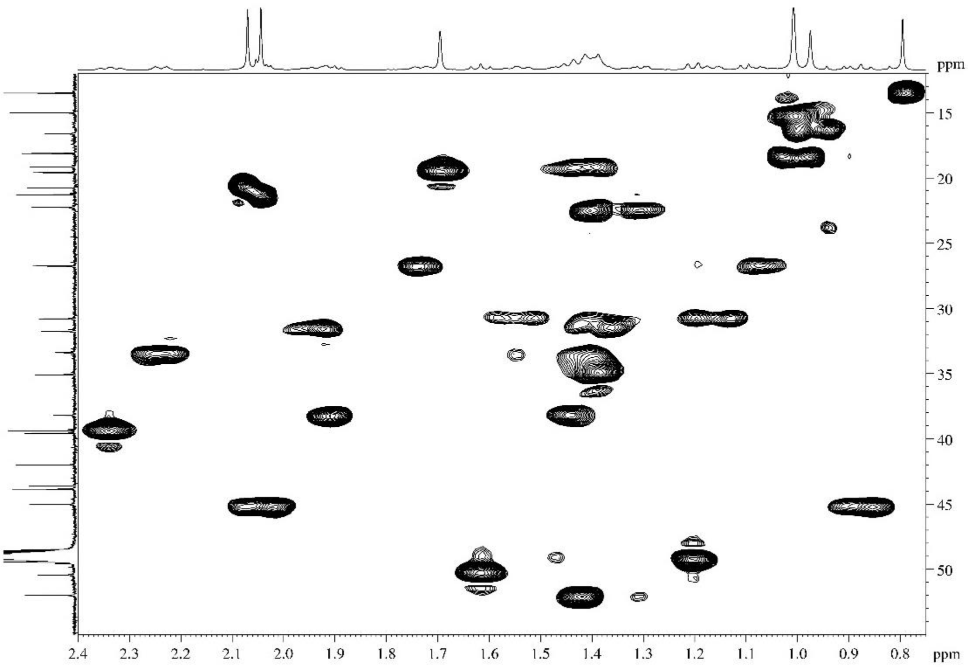

Figure S49. Enlarged HSQC (CD_3OD) spectrum of compound **5**

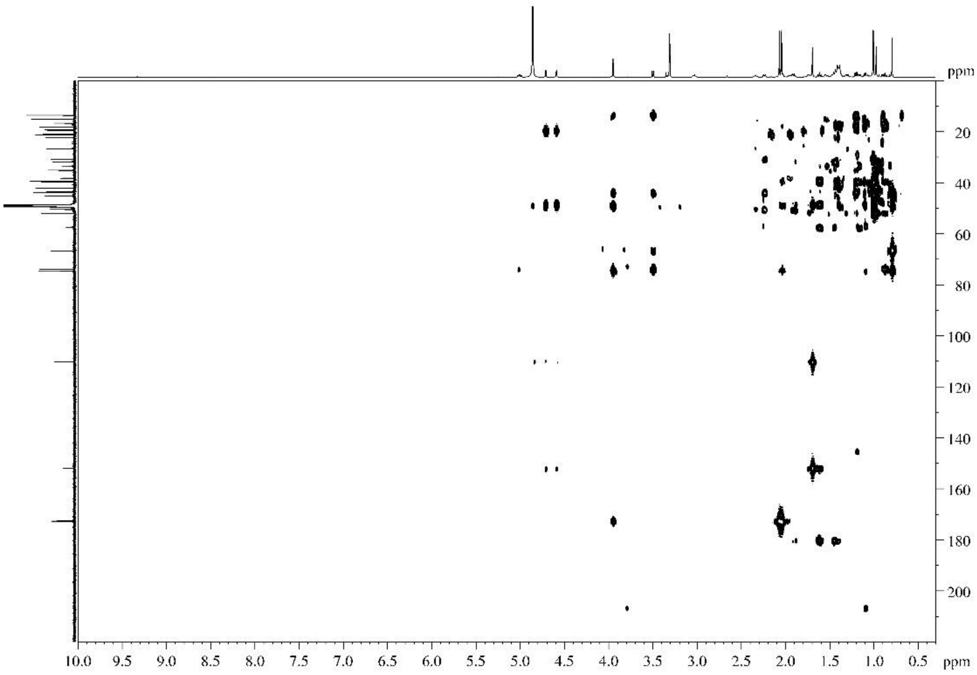

Figure S50. HMBC (CD_3OD) spectrum of compound **5**

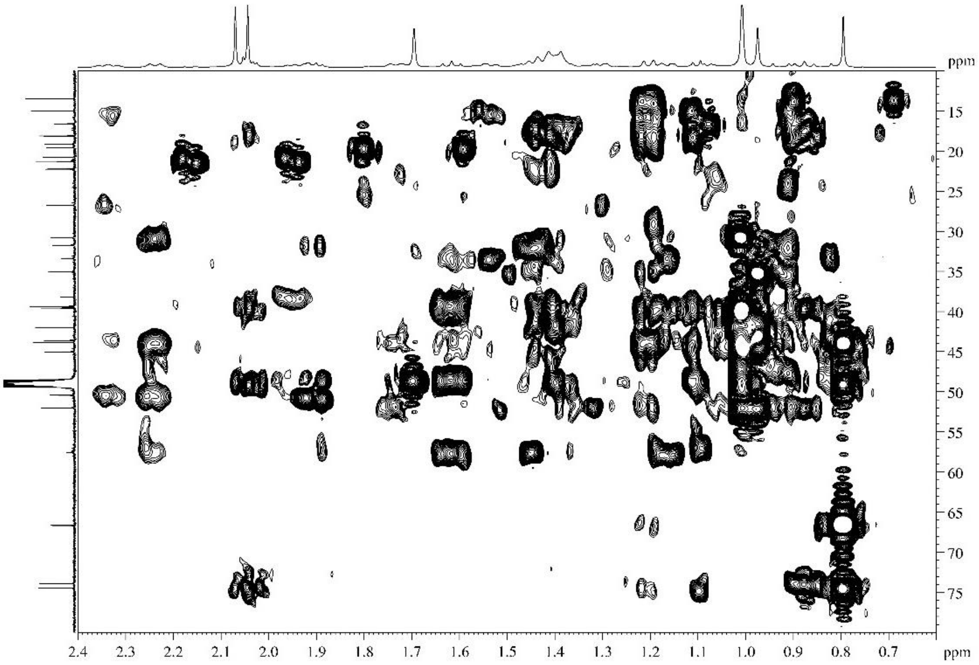

Figure S51. Enlarged HMBC (CD$_3$OD) spectrum of compound **5**

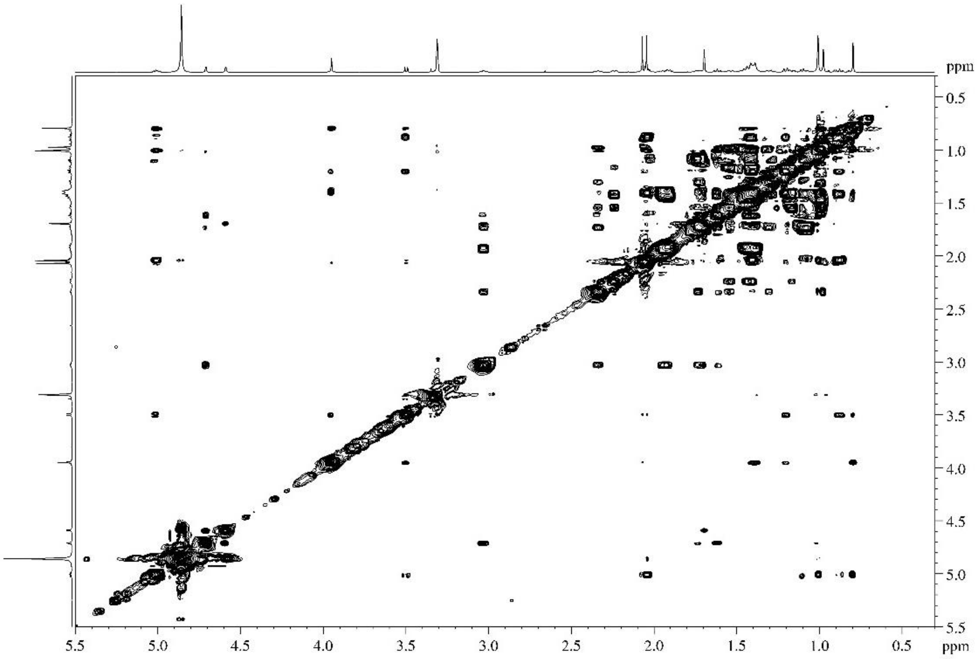

Figure S52. NOESY (CD$_3$OD) spectrum of compound **5**

Qualitative Compound Report

**Data File**	G-930-PXH-5.d	**Sample Name**	G-930-PXH-5
**Sample Type**	Sample	**Position**	P1-A6
**Instrument Name**	Instrument 1	**User Name**	
**Acq Method**	neg-1min.m	**Acquired Time**	10/1/2022 12:35:46 AM
**IRM Calibration Status**	Success	**DA Method**	QG-907.m
**Comment**			
**Sample Group**		**Info.**	
**Stream Name**	LC 1	**Acquisition SW Version**	6200 series TOF/6500 series Q-TOF B.08.00 (B8058.0)

Compound Table

Compound Label	RT	Mass	Abund	Formula	Tgt Mass	Diff (ppm)
Cpd 1: C34 H52 O7	0.133	572.3707	180199	C34 H52 O7	572.3713	-1.07

MS Zoomed Spectrum

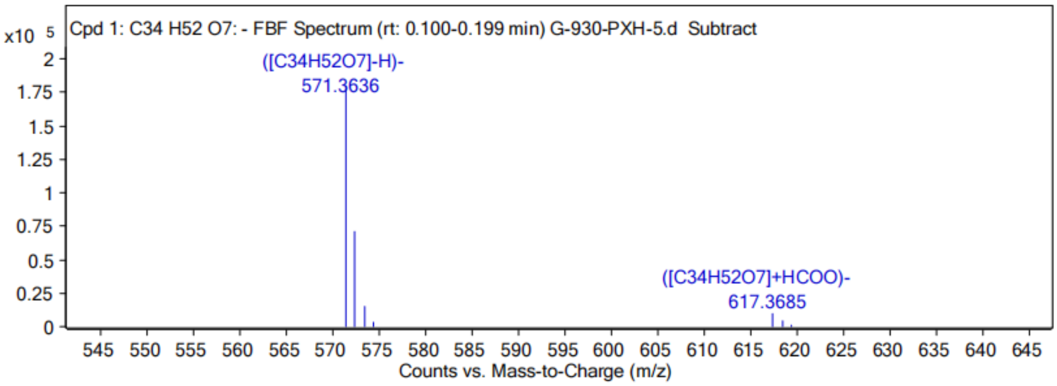

MS Spectrum Peak List

m/z	z	Abund	Formula	Ion
571.3636	1	180199.02	C34H52O7	(M-H)-
572.3667	1	70742.74	C34H52O7	(M-H)-
573.3694	1	15411.39	C34H52O7	(M-H)-
574.3691	1	2773.89	C34H52O7	(M-H)-
617.3685	1	9939.64	C34H52O7	(M+HCOO)-
618.3721	1	4033.8	C34H52O7	(M+HCOO)-
619.3739	1	1084.28	C34H52O7	(M+HCOO)-

--- End Of Report ---

Figure S53. HRESIMS spectrum of compound **5**

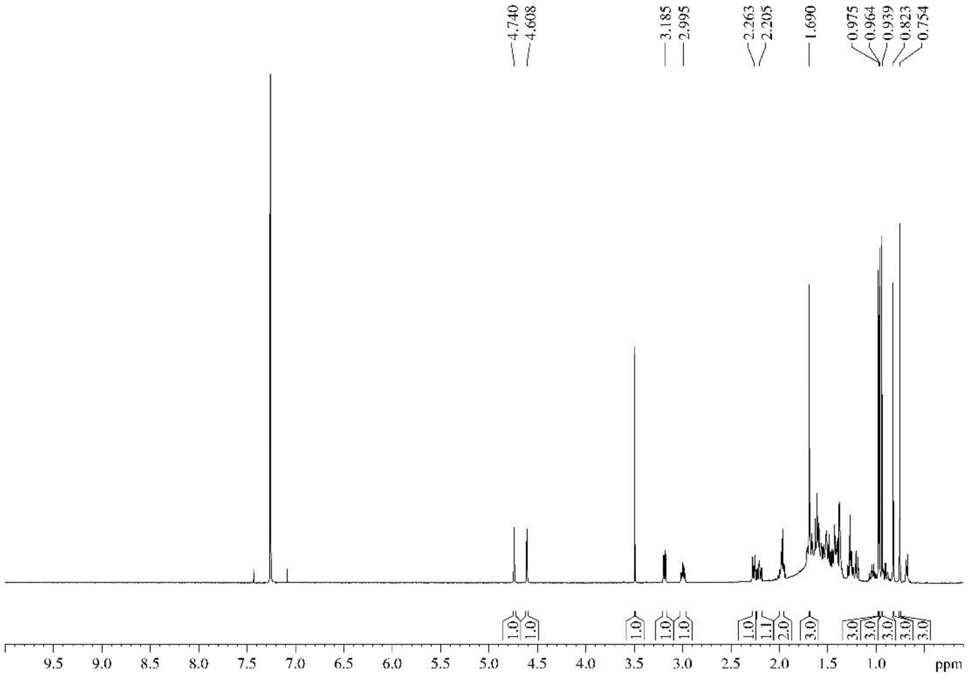

Figure S54. ^{1}H NMR (600 MHz, $CDCl_3$) spectrum of compound **6**

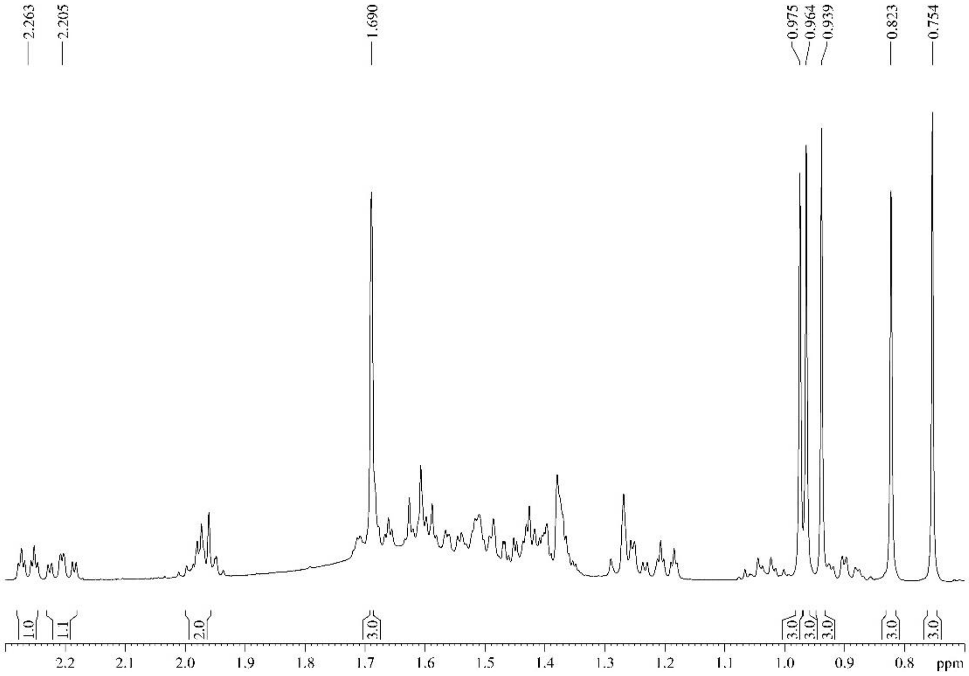

Figure S55. Enlarged ^{1}H NMR (600 MHz, $CDCl_3$) spectrum of compound **6**

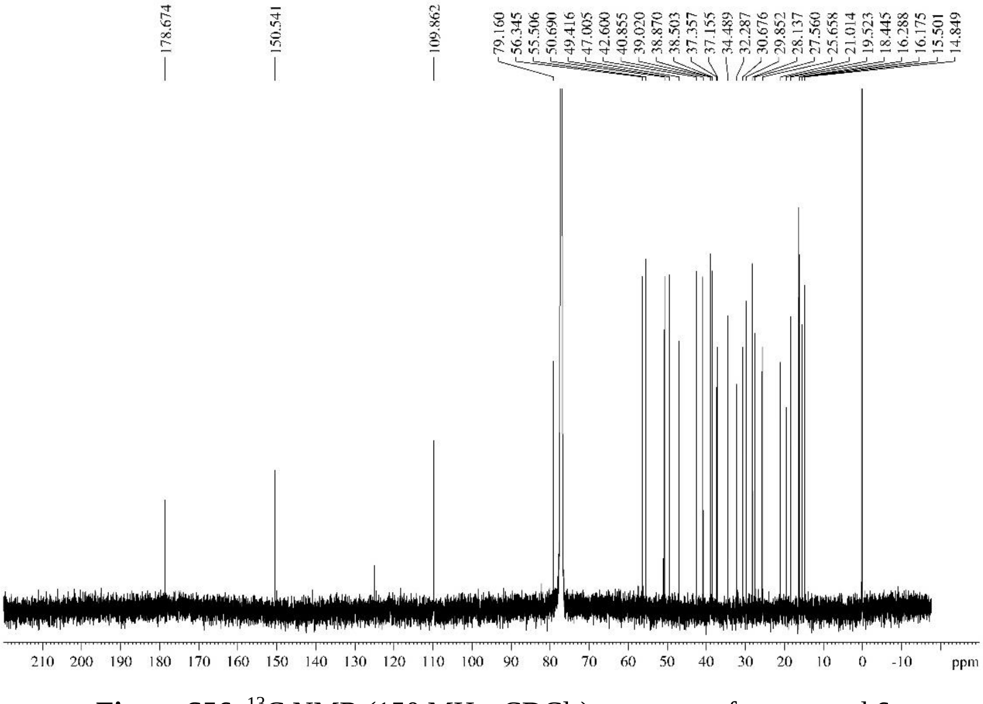

Figure S56. ^{13}C NMR (150 MHz, $CDCl_3$) spectrum of compound **6**

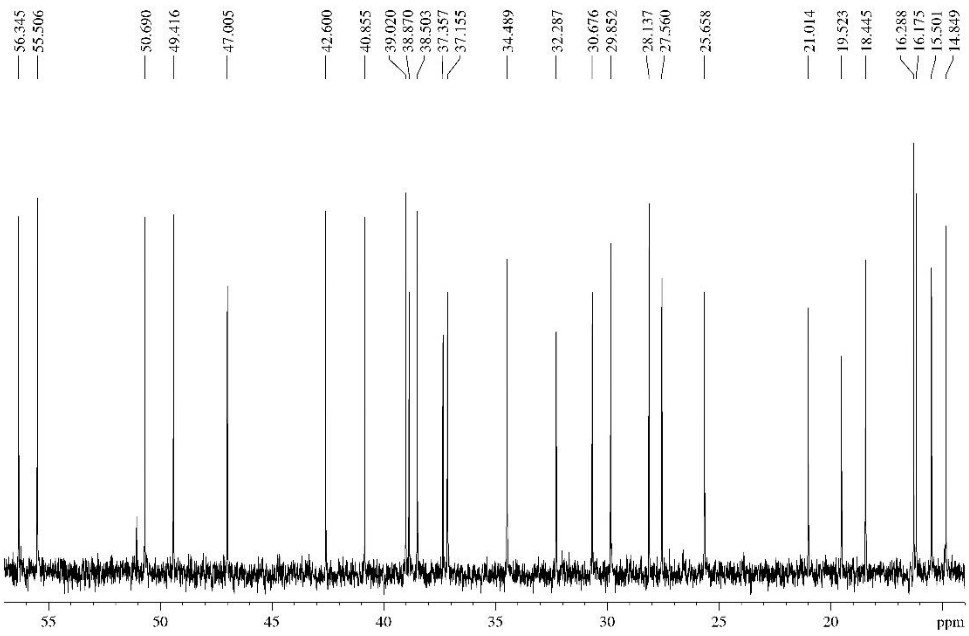

Figure S57. Enlarged ^{13}C NMR (150 MHz, $CDCl_3$) spectrum of compound **6**

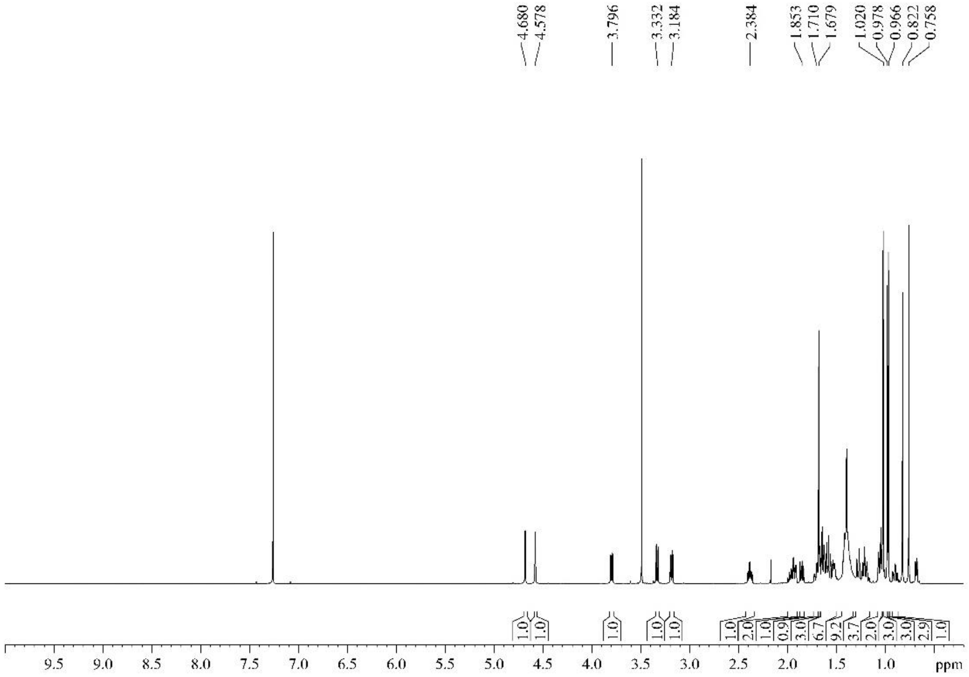

Figure S58. ^{1}H NMR (600 MHz, $CDCl_3$) spectrum of compound **7**

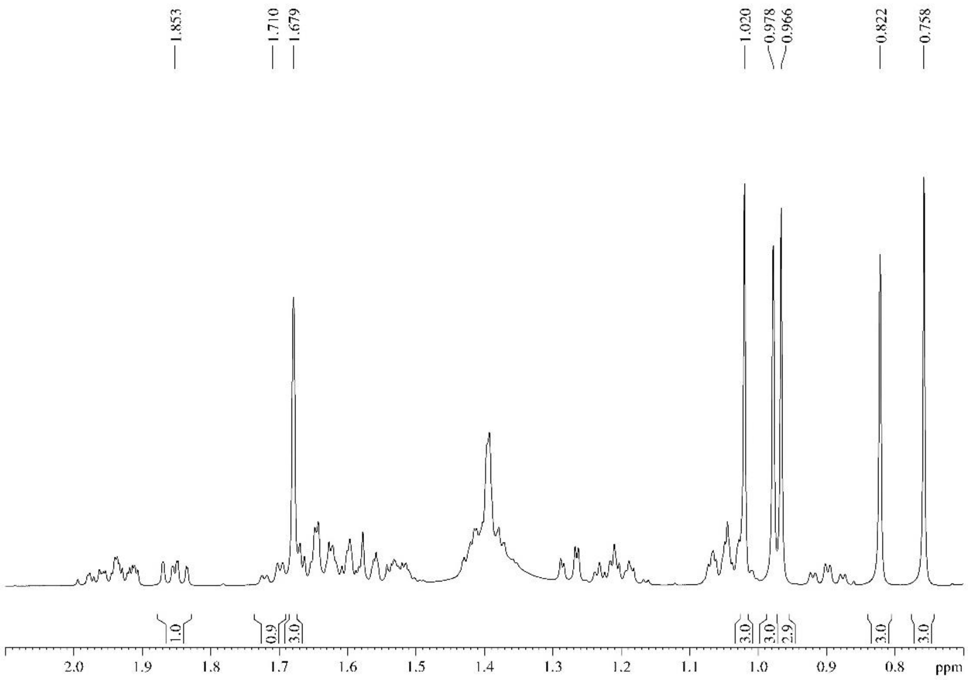

Figure S59. Enlarged ^{1}H NMR (600 MHz, $CDCl_3$) spectrum of compound **7**

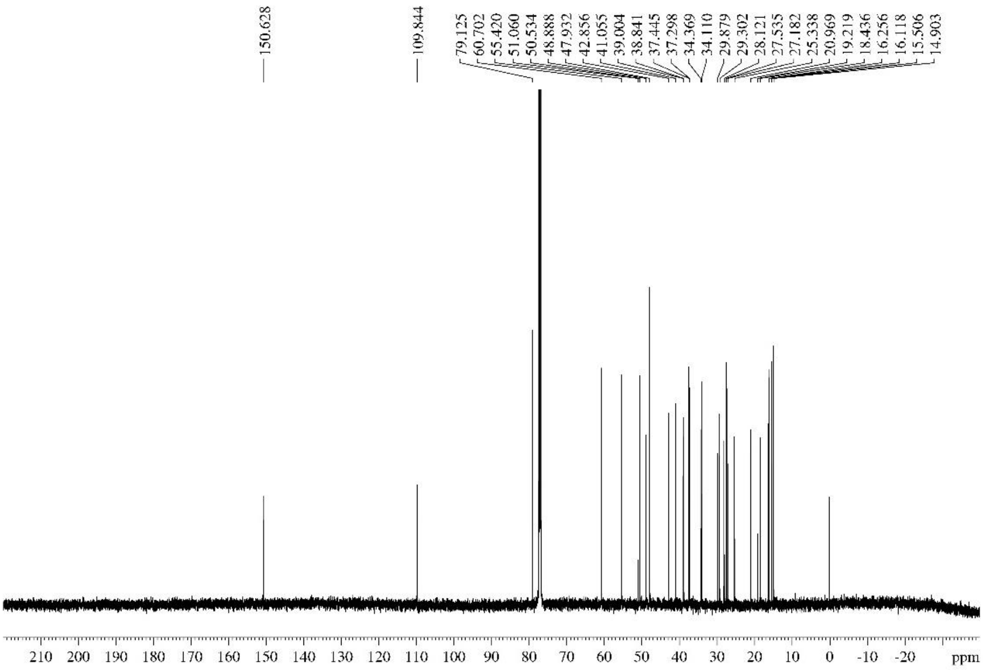

Figure S60. ^{13}C NMR (150 MHz, $CDCl_3$) spectrum of compound **7**

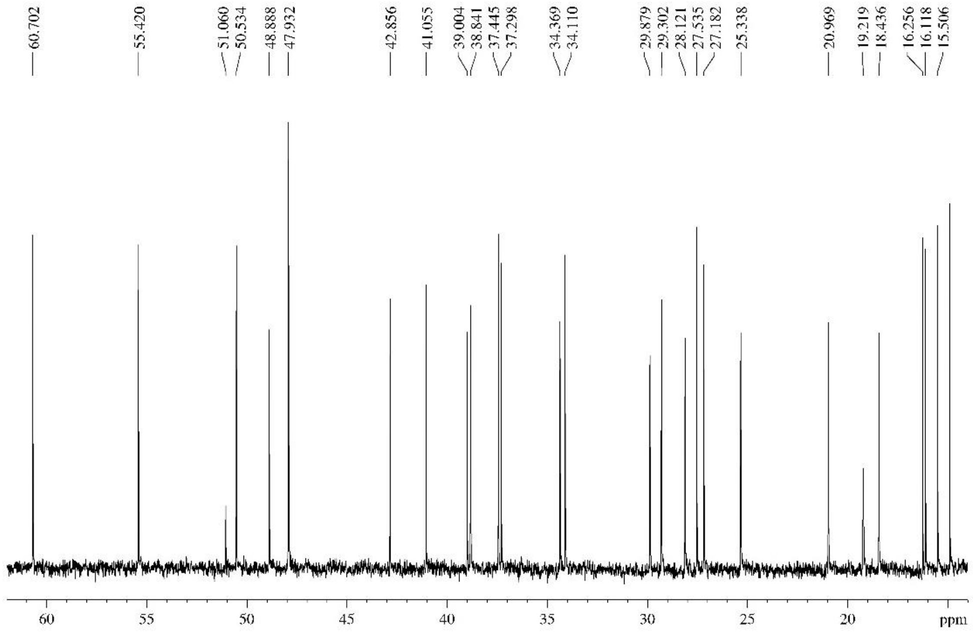

Figure S61. Enlarged ^{13}C NMR (150 MHz, $CDCl_3$) spectrum of compound **7**

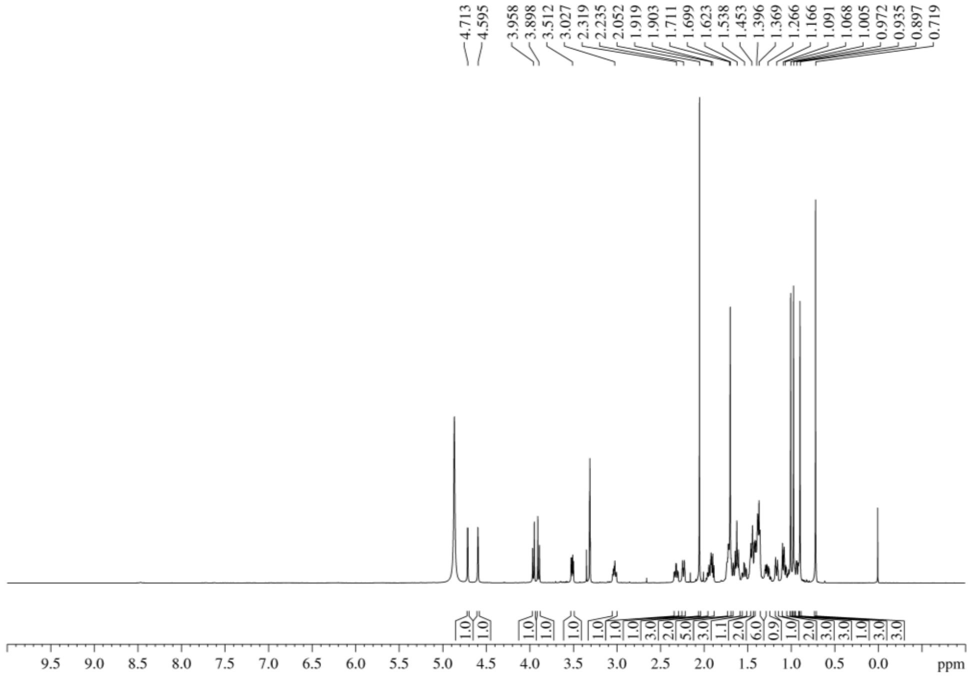

Figure S62. ^{1}H NMR (600 MHz, CD_3OD) spectrum of compound **8**

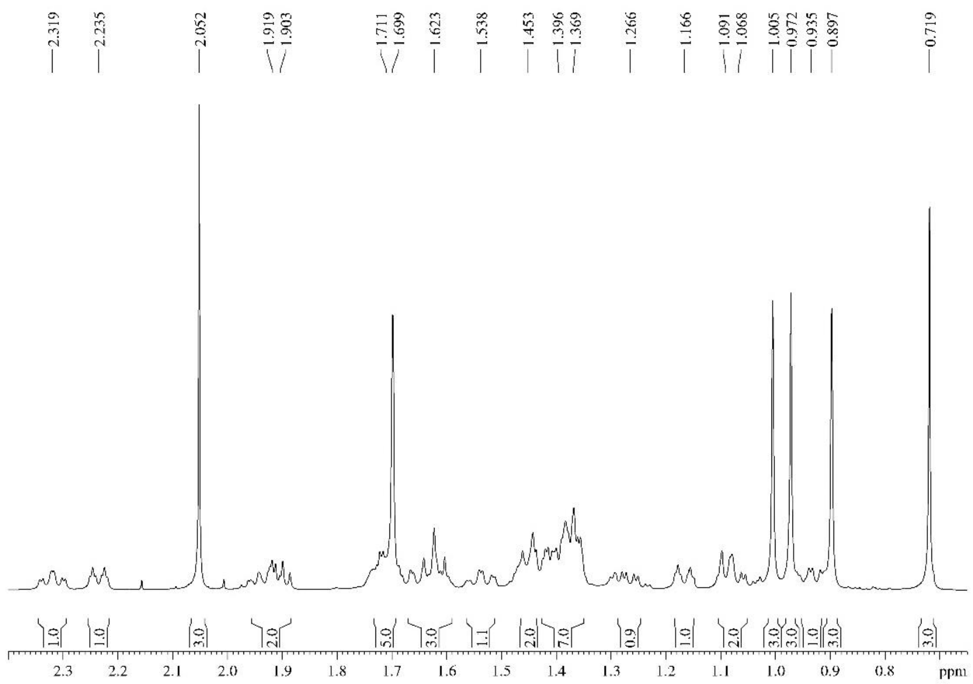

Figure S63. Enlarged ^{1}H NMR (600 MHz, CD_3OD) spectrum of compound **8**

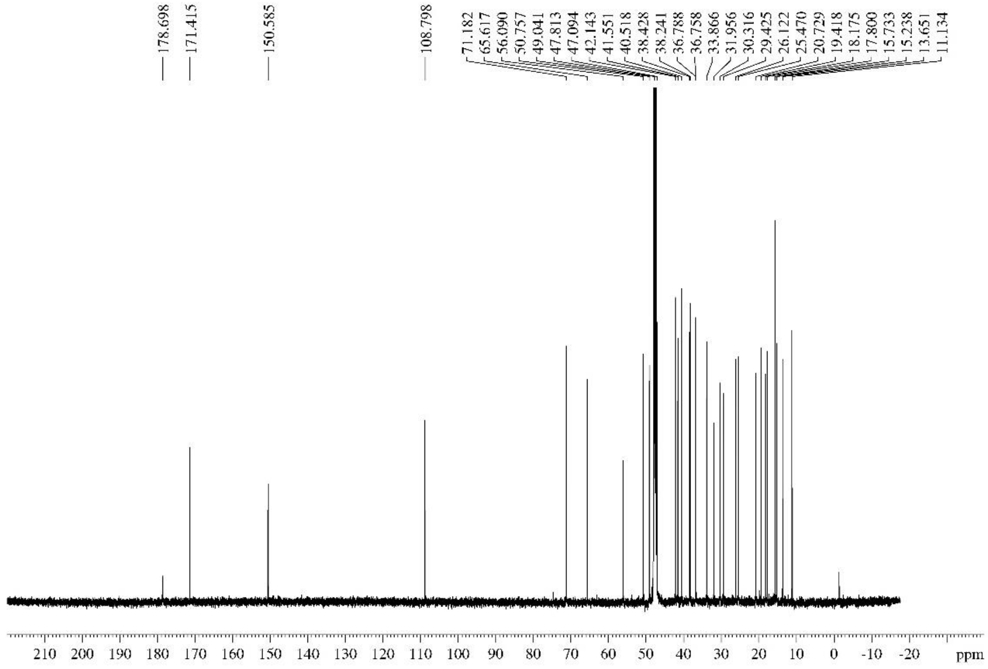

Figure S64. ^{13}C NMR (150 MHz, CD_3OD) spectrum of compound **8**

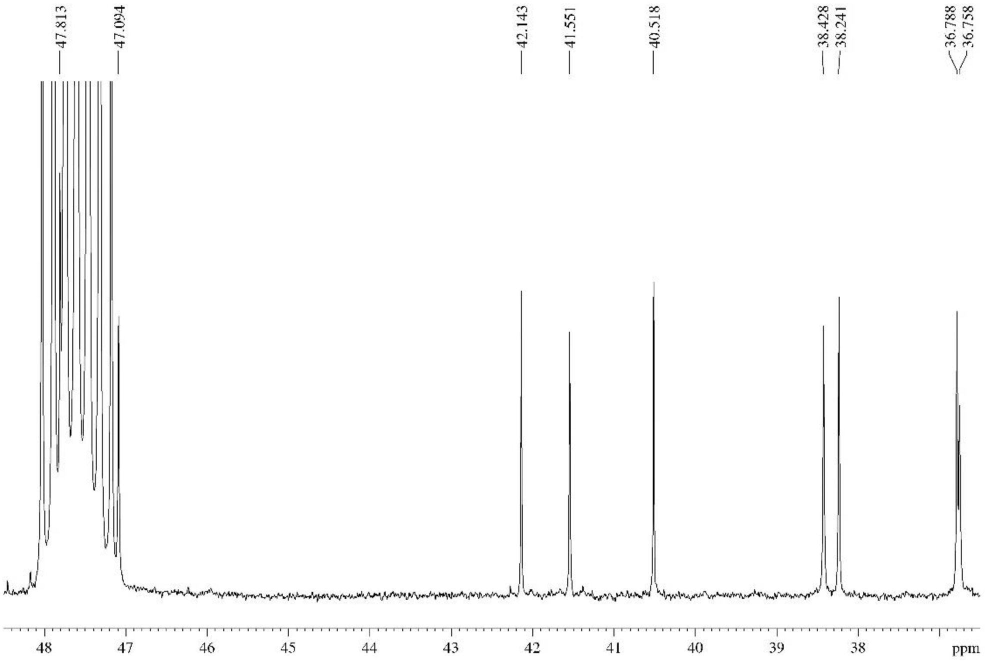

Figure S65. Enlarged ^{13}C NMR (150 MHz, CD_3OD) spectrum of compound **8**

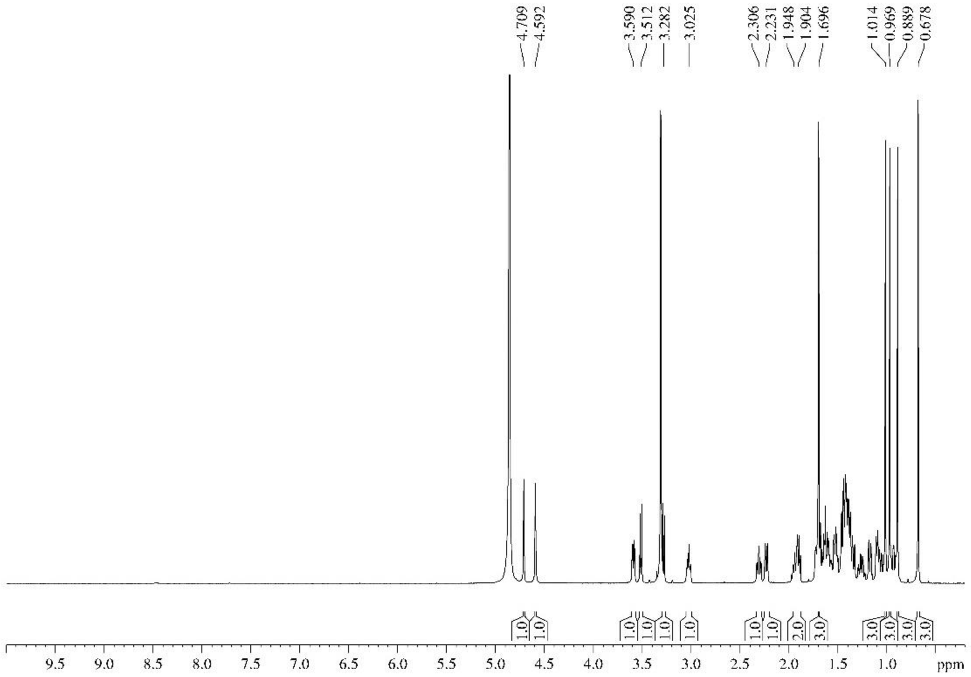

Figure S66. ^{1}H NMR (600 MHz, CD_3OD) spectrum of compound **9**

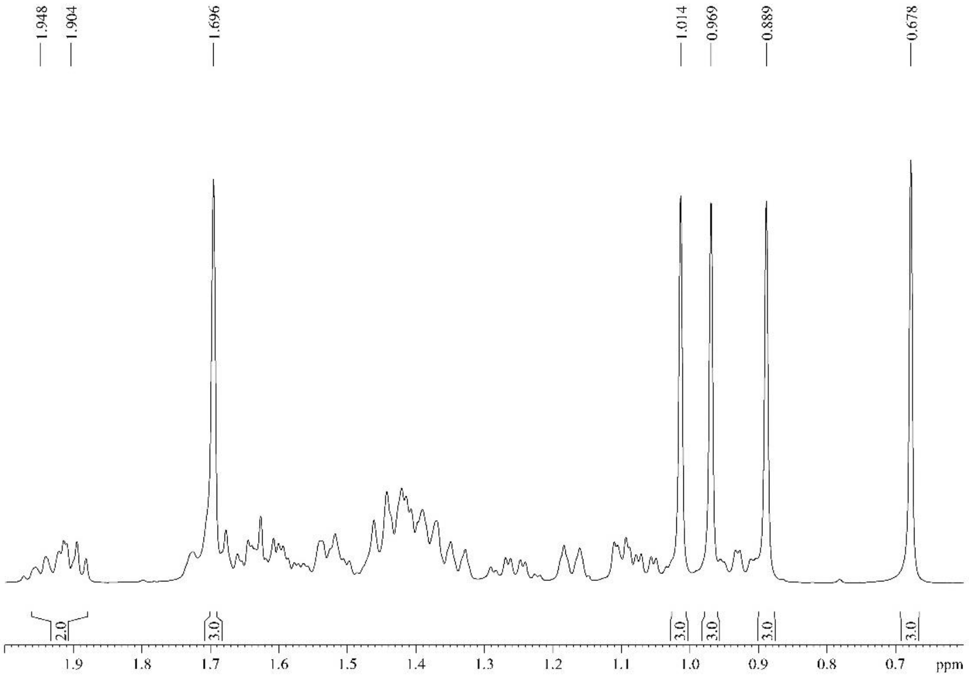

Figure S67. Enlarged ^{1}H NMR (600 MHz, CD_3OD) spectrum of compound **9**

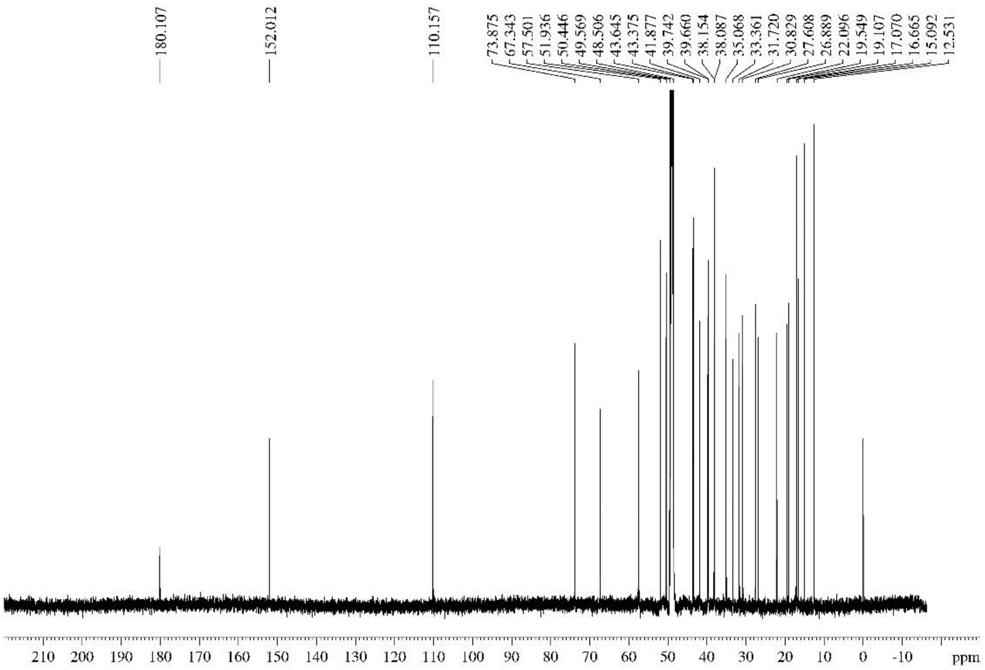

Figure S68. ^{13}C NMR (150 MHz, CD_3OD) spectrum of compound **9**

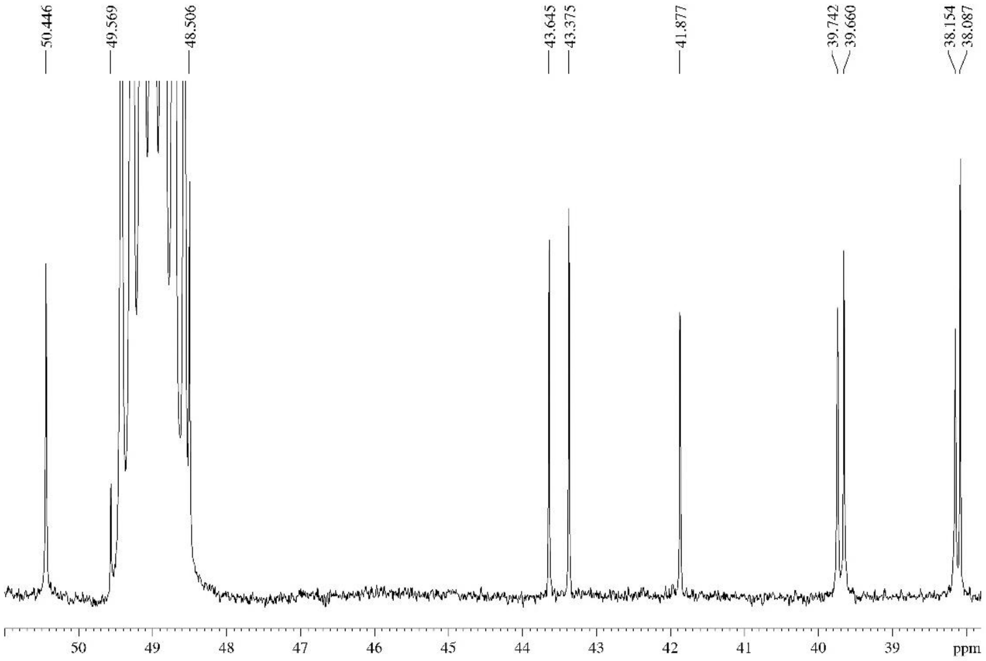

Figure S69. Enlarged ^{13}C NMR (150 MHz, CD_3OD) spectrum of compound **9**

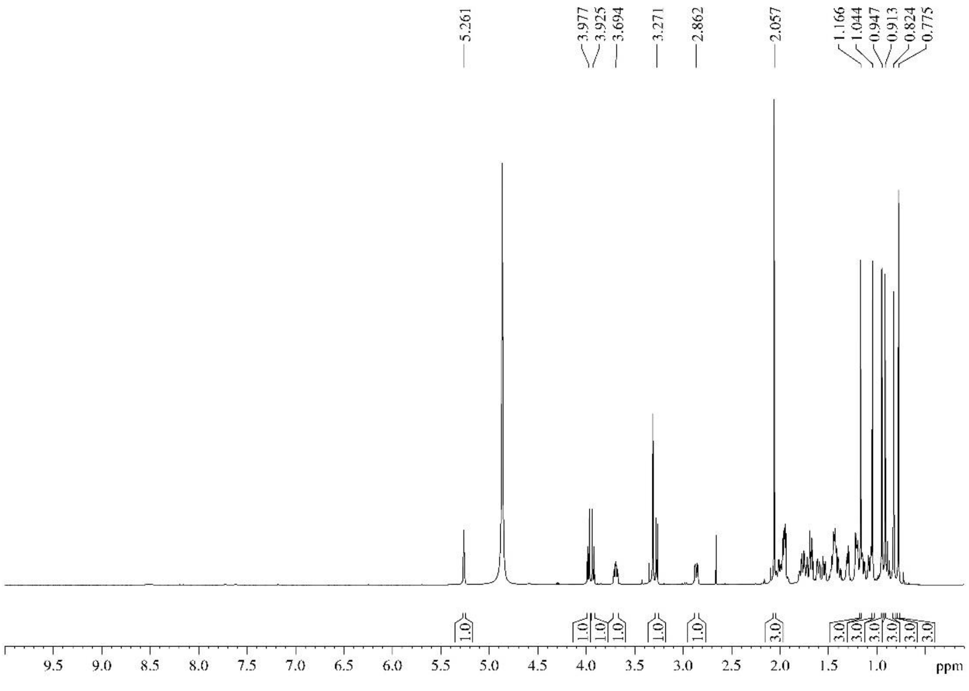

Figure S70. ^{1}H NMR (600 MHz, CD_3OD) spectrum of compound **10**

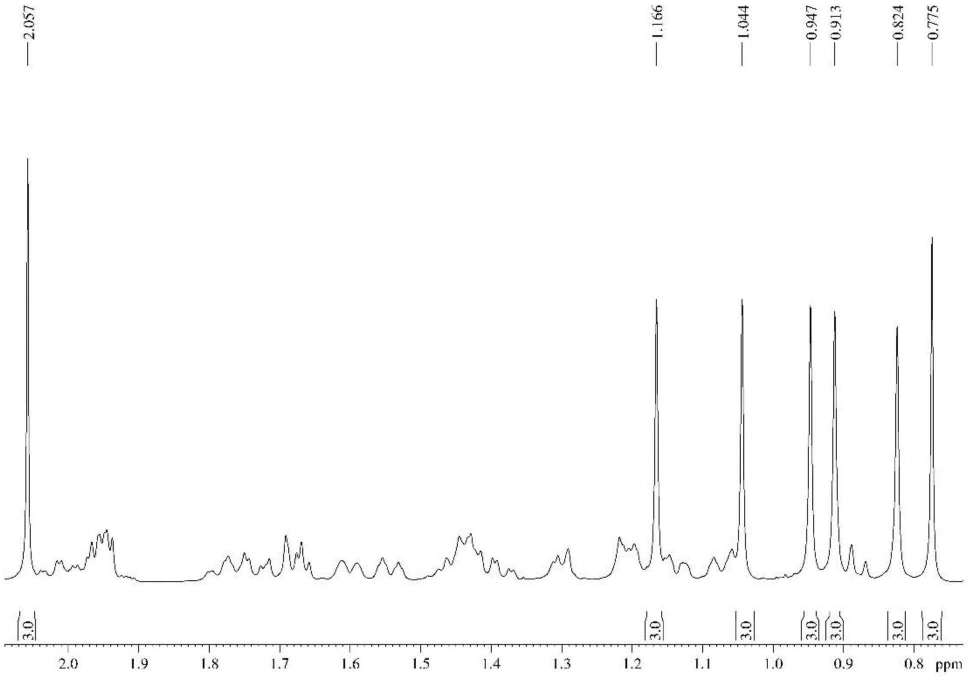

Figure S71. Enlarged ^{1}H NMR (600 MHz, CD_3OD) spectrum of compound **10**

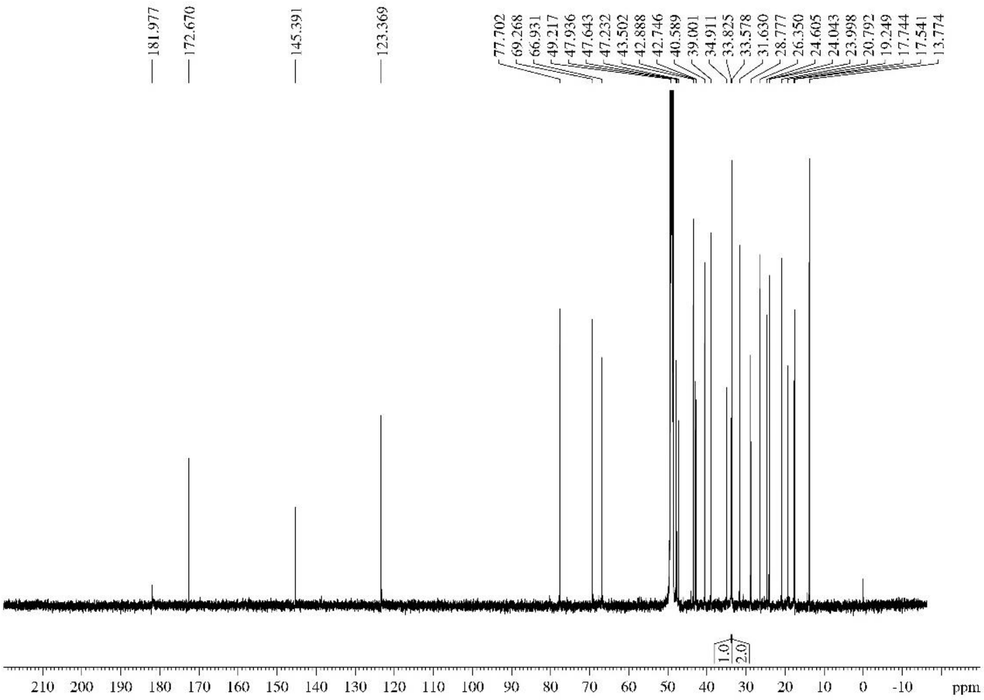

Figure S72. ^{13}C NMR (150 MHz, CD_3OD) spectrum of compound **10**

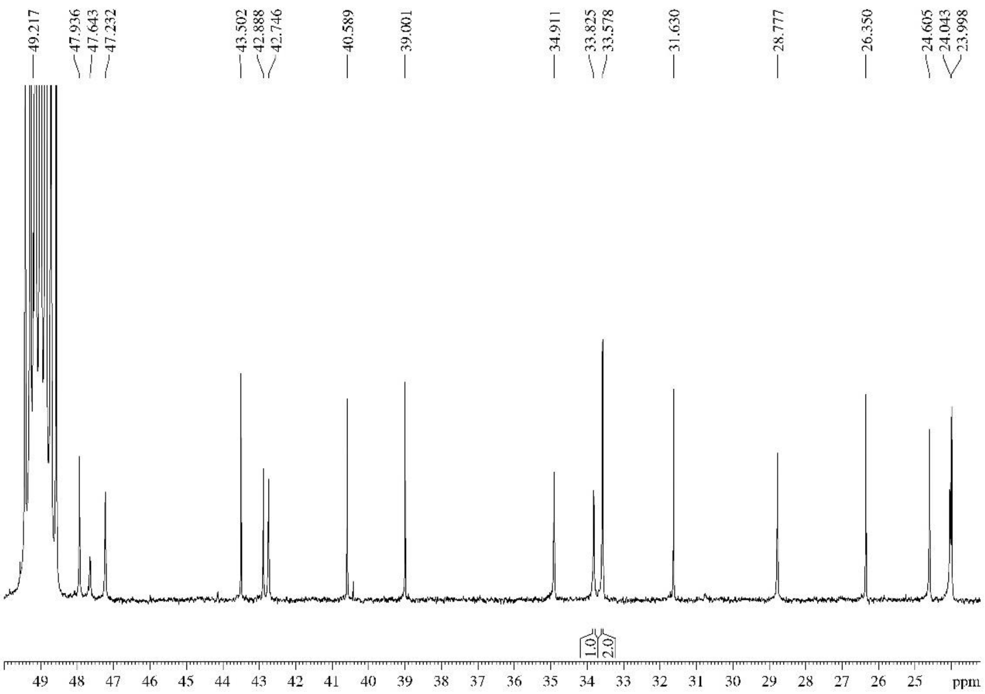

Figure S73. Enlarged ^{13}C NMR (150 MHz, CD_3OD) spectrum of compound **10**

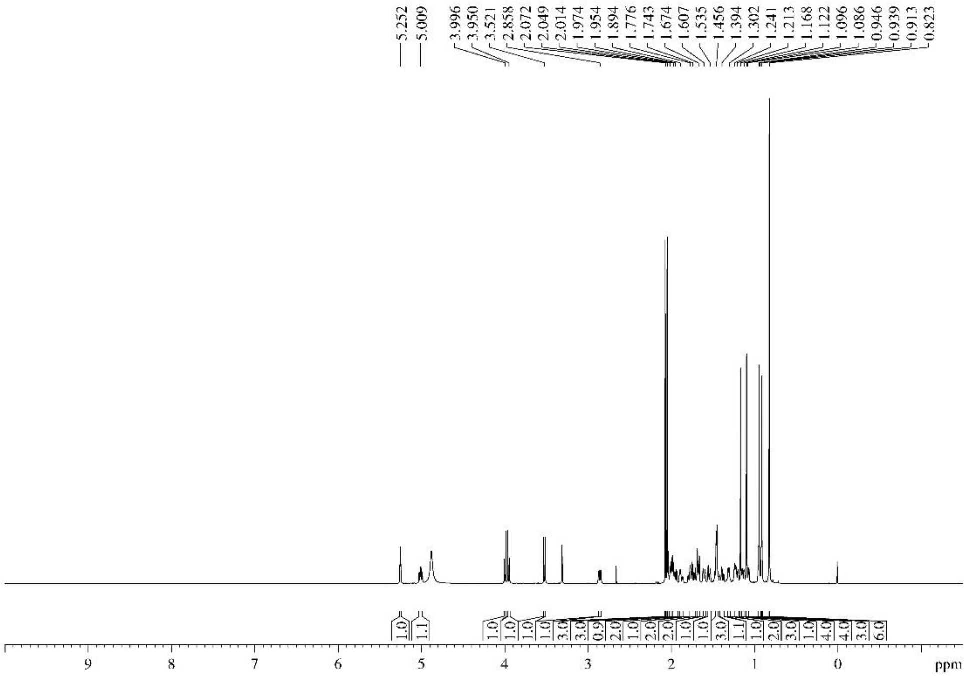

Figure S74. ^{1}H NMR (600 MHz, CD_3OD) spectrum of compound **11**

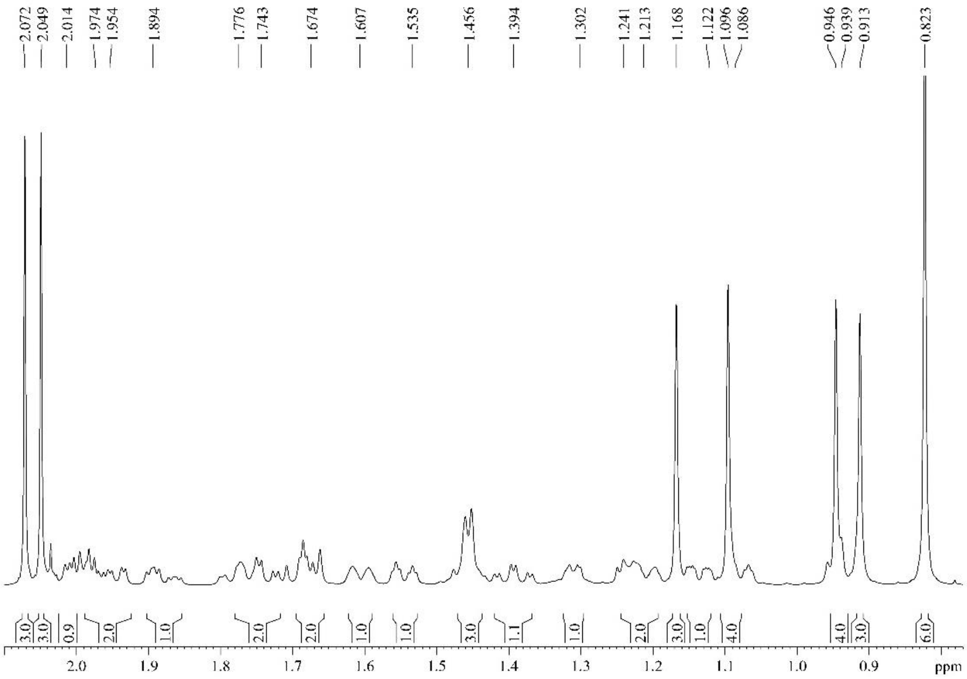

Figure S75. Enlarged ^{1}H NMR (600 MHz, CD_3OD) spectrum of compound **11**

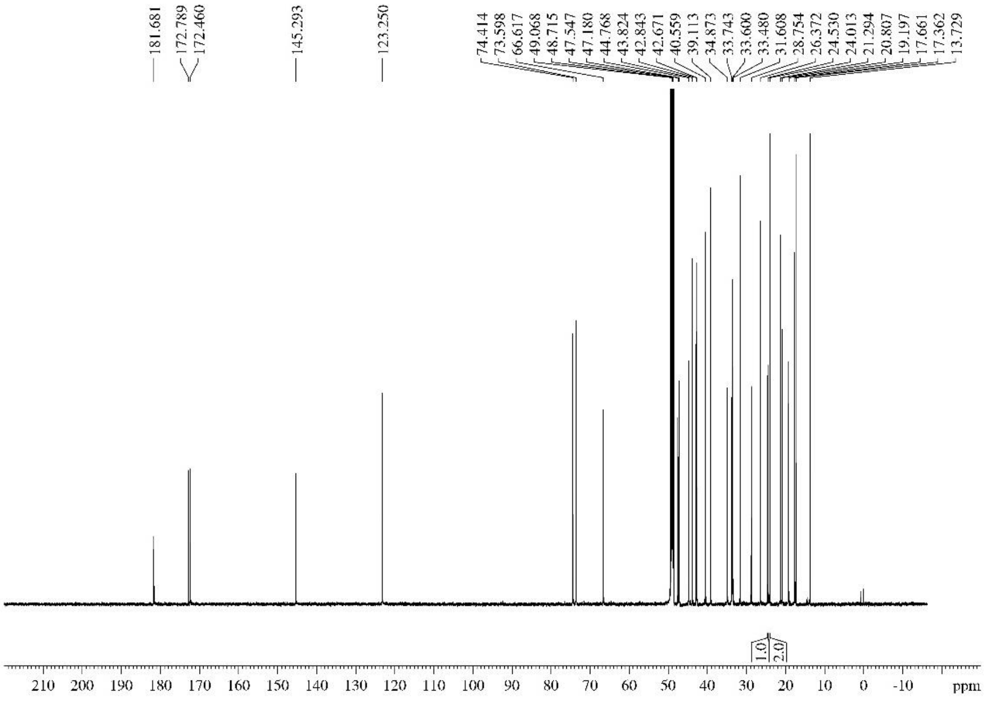

Figure S76. ^{13}C NMR (150 MHz, CD_3OD) spectrum of compound **11**

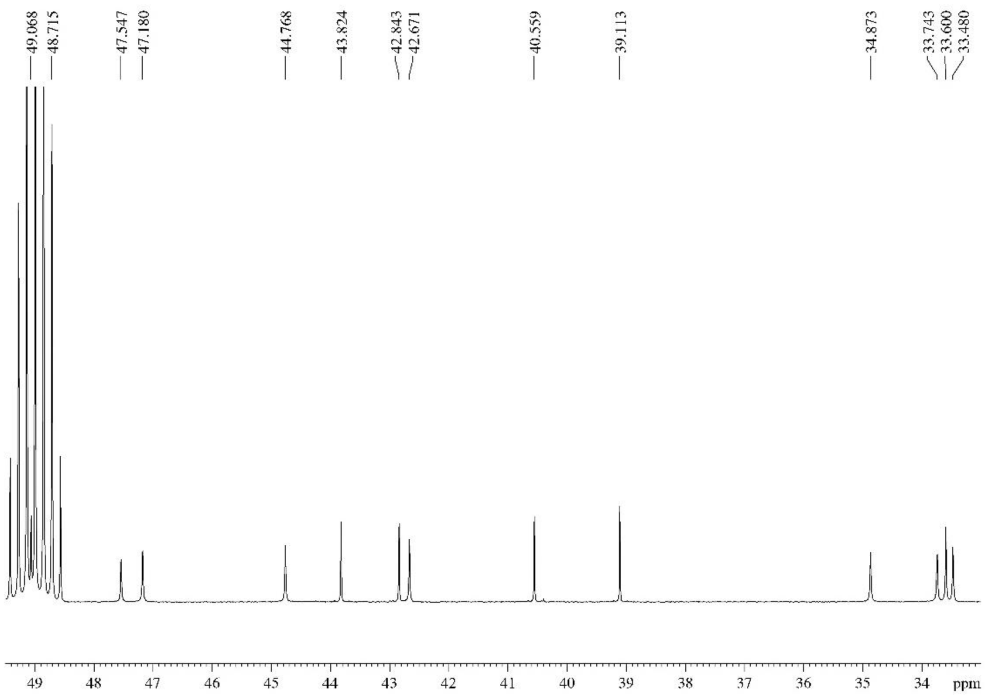

Figure S77. Enlarged ^{13}C NMR (150 MHz, CD_3OD) spectrum of compound **11**

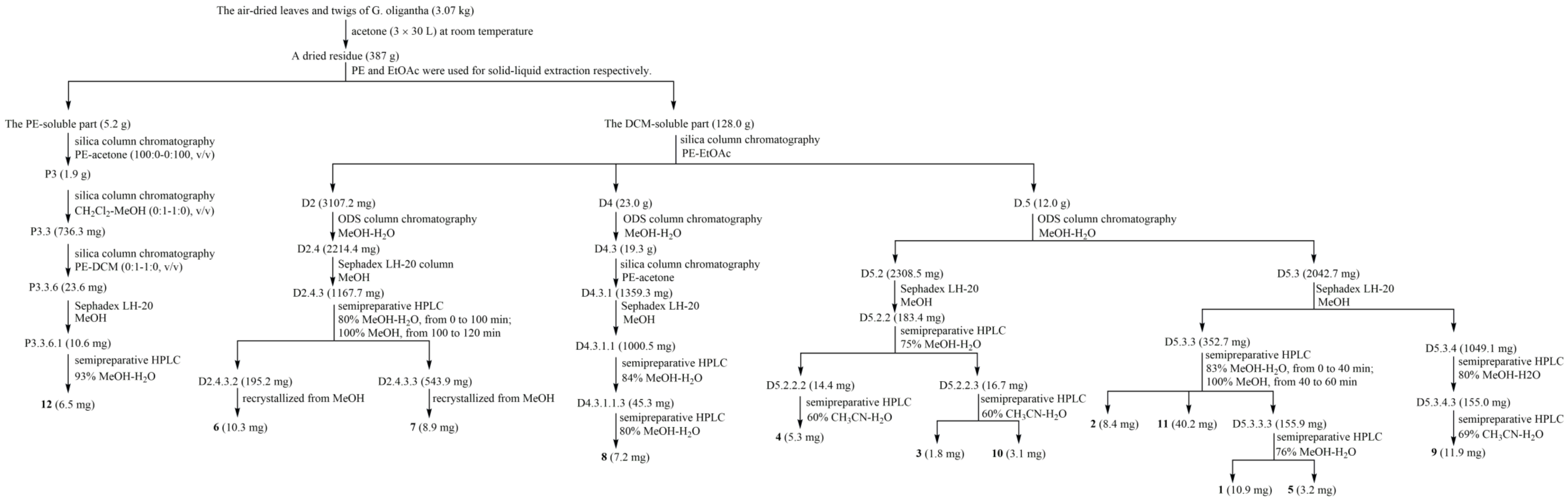

Figure S78. Extraction and separation flow chart
